# OHC-TRECK: A Novel System Using a Mouse Model for Investigation of the Molecular Mechanisms Associated with Outer Hair Cell Death in the Inner Ear

**DOI:** 10.1038/s41598-019-41711-2

**Published:** 2019-03-27

**Authors:** Kunie Matsuoka, Kenta Wada, Yuki Miyasaka, Shumpei P. Yasuda, Yuta Seki, Yasumasa Nishito, Hiromichi Yonekawa, Choji Taya, Hiroshi Shitara, Yoshiaki Kikkawa

**Affiliations:** 1 Mammalian Genetics Project, Tokyo Metropolitan Institute of Medical Science, 2-1-6 Kami-kitazawa, Setagaya-ku, Tokyo 156-8506 Japan; 2 Laboratory of Animal Resources and Development, Tokyo University of Agriculture, 196 Yasaka, Abashiri, Hokkaido, 099-2493 Japan; 3 Division of Experimental Animals, Nagoya University, 65 Tsurumai-cho Showa-ku, Nagoya, Aichi 466-8550 Japan; 4 Advanced Technical Support Department, Tokyo Metropolitan Institute of Medical Science, 2-1-6 Kami-kitazawa, Setagaya-ku, Tokyo 156-8506 Japan; 5 Laboratory for Transgenic Technology, Tokyo Metropolitan Institute of Medical Science, 2-1-6 Kami-kitazawa, Setagaya-ku, Tokyo 156-8506 Japan

## Abstract

Outer hair cells (OHCs) are responsible for the amplification of sound, and the death of these cells leads to hearing loss. Although the mechanisms for sound amplification and OHC death have been well investigated, the effects on the cochlea after OHC death are poorly understood. To study the consequences of OHC death, we established an OHC knockout system using a novel mouse model, *Prestin*-*hDTR*, which uses the prestin promoter to express the human diphtheria toxin (DT) receptor gene (*hDTR*). Administration of DT to adult *Prestin*-*hDTR* mice results in the depletion of almost all OHCs without significant damage to other cochlear and vestibular cells, suggesting that this system is an effective tool for the analysis of how other cells in the cochlea and vestibula are affected after OHC death. To evaluate the changes in the cochlea after OHC death, we performed differential gene expression analysis between the untreated and DT-treated groups of wild-type and *Prestin*-*hDTR* mice. This analysis revealed that genes associated with inflammatory/immune responses were significantly upregulated. Moreover, we found that several genes linked to hearing loss were strongly downregulated by OHC death. Together, these results suggest that this OHC knockout system is a useful tool to identify biomarkers associated with OHC death.

## Introduction

Sound waves deflect stereocilia bundles on the apical surfaces of hair cells to activate mechanoelectrical transduction channels, which causes depolarization and activation of afferent neurons^1–3^. There are two types of hair cells in the mammalian cochlea, namely, inner hair cells (IHCs) and outer hair cells (OHCs). IHCs are conventional sensory receptors that transmit most of the acoustic information to the brain via ribbon synapses and type I spiral ganglion neurons (SGNs), which represent 90–95% of all SGNs, forming auditory afferent fibers^1–3^. In contrast, OHCs are mainly innervated by efferent fibers and contact with type II SGNs, which constitute only 5–10% of all SGNs^1–3^. However, OHCs play an important role in enhancing the sensitivity to sound. The main task of OHCs is the amplification of sound-induced vibrations via two mechanical activities, hair bundle motility and somatic motility^1,2,4^.

Genetic and environmental risk factors often damage hair cells and lead to the death of these cells. Previous studies have reported that OHCs are more susceptible to environmental risk factors than IHCs based on experiments on animal models. Noise exposure rapidly induces loss of OHCs compared to IHCs in mice^5–7^, guinea pigs^8^, and chinchillas^9^. The susceptibility to ototoxic drugs, antineoplastic agents (such as cisplatin), and aminoglycoside antibiotics (such as kanamycin and gentamicin) also differs between IHCs and OHCs. Although these drugs induce apoptotic cell death of both IHCs and OHCs via abnormal accumulation of reactive oxygen species and oxidative stress^1,10^, OHC loss occurs earlier and more extensively than IHC loss in mice^11–13^, rats^13^, guinea pigs^14^, and hamsters^15^. In addition, age-related damage is more extensive in OHCs than in IHCs. Several studies have reported that progressive OHC loss was more severe than IHC loss in aged mice^10,16–18^.

Studies to elucidate the molecular mechanisms underlying the protection of OHCs from death and the regeneration of OHCs are important because OHC death is associated with several types of hearing loss. Although noise exposure, administration of ototoxic drugs and aged animals have been used as experimental tools to induce degeneration and loss of OHCs, each of these models has some limitations that restrict its use in OHC studies. OHC death is difficult to induce effectively via noise exposure and administration of ototoxic drugs, and researchers must wait for long periods for OHC loss to occur in aged mice. Moreover, the major and most common problem associated with these experimental models is that damage is caused not only to OHCs but also to other cells and tissues, such as afferent fibers or synapses^19–21^, the stria vascularis/lateral wall^6,17^ and vestibular hair cells (VHCs)^12^. Therefore, new animal models are needed to selectively deplete OHCs without any damage to the other cochlear and vestibular cells to investigate the effect of OHC death on these other cells.

Here, we report on the OHC-toxin receptor-mediated conditional cell knockout (TRECK) system, which enables selective depletion of OHCs. The TRECK method involves conditional depletion of a target cell *in vivo* via administration of diphtheria toxin (DT) to transgenic (tg) mice carrying a human DT receptor (*hDTR*) cDNA transgene under the control of a tissue-specific promoter^22,23^. In this study, we show that the use of the OHC-TRECK system with a novel mouse model, *Prestin*-*hDTR*, which carries the prestin promoter and the *hDTR* gene, induces selective depletion of OHCs upon DT administration. Therefore, this mouse model could be useful in studies pertaining to OHC death, although the mechanisms and processes underlying OHC death here would differ from those in mouse models generated by noise exposure, ototoxic drugs, and aging.

## Results

### Generation of *Prestin*-*hDTR* mice

**Figure 1:**
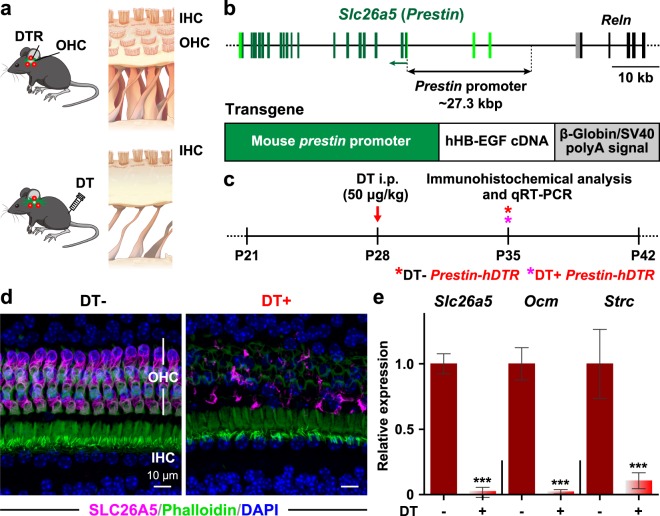
Generation of *Prestin*-*hDTR* mice. (**a**) Schematic representation of the selective and inducible depletion of OHCs using *Prestin*-*hDTR* mice and administration of DT. 2019 by Emiko Wakatsuki. Used with permission. (**b**) Transgene construct for *Prestin*-*hDTR* mice. The genomic structure of the longest transcript (Ensemble transcript ID: ENSMUST00000030878.7) in *Slc26a5*/*Prestin* is shown on top with *Reln* (reelin gene) located adjacent to the distal region. The dark- and light-green boxes indicate coding and noncoding regions, respectively, in 20 exons of *Slc26a5*. The green arrow shows the direction of transcription. An illustration of the transgene construct is shown at the bottom. The construct was generated by ligation of the *Slc26a5* promoter (green box), modified *hHB-EGF* (I117V/L148V) (white box), *β-globin* and SV40 pA^+^ (gray box). (**c**) Schematic representation of the experimental design for confirmation of OHC-TRECK in *Prestin*-*hDTR* mice after administration of DT. *Prestin*-*hDTR* mice at P28 were treated with DT at 50 μg/kg (red arrow), and tissue samples were collected for immunohistochemical analysis (**d**) and qRT-PCR analysis (**e**) at day 7 (P35) (asterisks) after administration of DT (DT+) by comparison of the RNA levels and expression of Prestin (SLC26A5) with those in the OHCs of untreated (DT−) *Prestin*-*hDTR* littermates. (**d**) Confocal images of SLC26A5/prestin (magenta) in cochlear hair cells from DT− and DT+ *Prestin*-*hDTR* mice. The cochleae were counterstained with phalloidin (green) and DAPI (blue). IHC: inner hair cell. (**e**) qRT-PCR analysis of the OHC markers *Slc26a5*, oncomodulin (*Ocm*), and stereocilin (*Strc*) in cochlear RNA of DT− (*n* = 3) and DT+ (*n* = 6) *Prestin*-*hDTR* mice. Asterisks indicate statistical significance: ***P < 0.001 (Student’s t-test). Error bars = standard deviations (SDs).

The goal of our study was to selectively deplete OHCs *in vivo* using the TRECK system (Fig. 1a). A genomic region including the promoter and noncoding exons 1 and 2 of murine *Slc26a5* (solute carrier family 26, member 5 gene, also known as prestin), which is targeted to the lateral walls of OHCs^24^, was used to drive the expression of human heparin-binding EGF-like growth factor (*hHB-EGF*) as a DT receptor (Fig. 1b). The transgene also contained the polyadenylation signals (pA^+^) of rabbit *β-globin* and simian virus 40 (SV40). *hHB*-*EGF* was modified at two amino acids (I117V and L148V) within the EGF-like domain. Furukawa *et al*. reported that modification of *hHB*-*EGF* reduces the side effects of EGF-like growth factor activity, such as phosphorylation of the EGF receptor and transduction of signals to neighboring cells, while maintaining the DT sensitivity of wild-type *hHB*-*EGF*^25^. We microinjected the construct into fertilized eggs from C57BL/6J mice and confirmed the integration of the transgene into five mice, and three founders exhibited germline transmission. We administered DT to mice from these three tg lines (#38, #53, and #65) at postnatal days 28 (P28) by intraperitoneal (i.p.) injection at a dose of 50 μg/kg and analyzed the OHC phenotype and gene expression levels by immunohistochemistry and quantitative RT-PCR (qRT-PCR) after 7 days, as shown in Fig. 1c. The #38 tg mice exhibited the most severe phenotypes after DT administration. The OHC-specific protein expression of SLC26A5/prestin in DT-treated *Prestin*-*hDTR* #38 mice was nearly abolished (Fig. 1d). The expression levels of the OHC marker genes *Slc26a5*; *Ocm* (oncomodulin), which is highly expressed in the cytoplasm of OHCs^26^; and *Strc* (stereocilin), which is localized in the horizontal top connectors of OHC hair bundles^27,28^, were dramatically decreased to nearly undetectable levels in the cochlear mRNA of DT-treated *Prestin*-*hDTR* #38 mice compared with the expression levels of these genes in untreated *Prestin*-*hDTR* mice (Fig. 1e). Moreover, by ligation-mediated PCR, we confirmed that the transgene in the #38 mice was integrated into the E3 region on chromosome 14 (Supplementary Fig. S1). There are no known coding or noncoding genes in the vicinity of the integration site. Therefore, we selected the #38 line as a suitable *Prestin*-*hDTR* mouse model for further experiments.

### *In vivo* OHC depletion by OHC-TRECK

**Figure 2:**
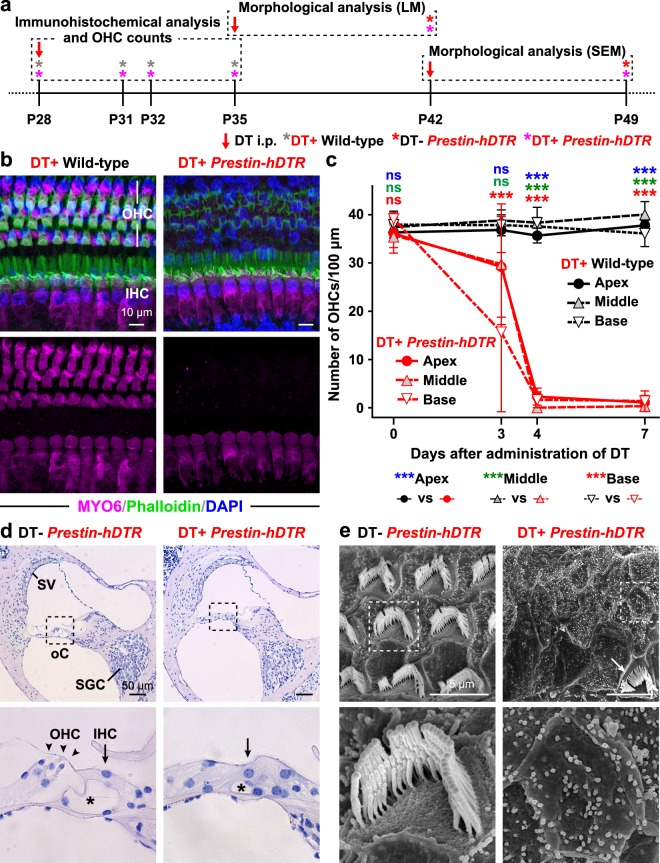
Inducible depletion of OHCs in *Prestin*-*hDTR* mice by administration of DT. (**a**) Timelines of experiments and DT administration for immunohistochemical analysis, OHC counts, morphological analysis of cochleae and OHCs using light microscopy (LM) and scanning electron microscopy (SEM). Red arrows and asterisks indicate the days of DT administration and tissue correction for each experiment. (**b**) Confocal images of the hair cells from the middle area of the cochlea in DT-treated (DT+) wild-type and *Prestin*-*hDTR* mice. The cochleae of mice were stained with anti-myosin VI (MYO6) antibody (magenta), phalloidin (green), and DAPI (blue). (**c**) OHC counts in the apical (A), middle (M), and basal (B) areas of the cochleae of DT+ wild-type (WT) and *Prestin*-*hDTR* (*P*-*hDTR*) mice at 0 (area A, *n* = 19 WT/7 *P*-*hDTR*; area M, *n* = 17 WT/8 *P*-*hDTR*; area B, *n* = 28 WT/16 *P*-*hDTR*), 3 (area A, *n* = 6 WT/31 *P*-*hDTR*; area M, *n* = 5 WT/26 *P*-*hDTR*; area B, *n* = 6 WT/19 *P*-*hDTR*), 4 (area A, *n* = 3 WT/12 *P*-*hDTR*; area M, *n* = 3 WT/5 *P*-*hDTR*; area B, *n* = 3 WT/6 *P*-*hDTR*), and 7 (area A, *n* = 9 WT/9 DT+ OT; area M, *n* = 8 WT/9 *P*-*hDTR*; area B, *n* = 9 WT/8 *P*-*hDTR*) days after administration of DT. The means (circles and triangles) and SDs (error bars) of the OHC counts are shown for each mouse. Asterisks indicate statistical significance: *P < 0.05 and ***P < 0.001 (two-way ANOVA with Bonferroni post-hoc comparison for the data from all days for each mouse). ns, no significant difference. (**d**) Hematoxylin staining of sections from the middle area of the cochleae of untreated (DT−) and DT+ *Prestin*-*hDTR* mice. High-magnification images of the organ of Corti (oC), indicated by dotted boxes in the top panels, are shown in the bottom panels. Asterisks indicate the tunnel of Corti. SV, stria vascularis and SGC, spiral ganglion cell. (**e**) SEM images of OHCs from the middle area of the cochleae in the DT− and DT+ *Prestin*-*hDTR* mice. High-magnification images of the OHCs indicated by dotted boxes in the top panels are shown in the bottom panels. The arrow indicates an OHC that retained stereocilia in DT+ *Prestin*-*hDTR* mice.

First, we investigated the phenotypes of the OHCs by OHC-TRECK of *Prestin*-*hDTR* mice at P28 using immunohistochemical and histopathological analyses (Fig. 2a). By staining for myosin VI (MYO6), a known hair cell marker^18,29^, we confirmed that OHCs from *Prestin*-*hDTR* mice were almost all lost from the organ of Corti 7 days after administration of DT. Strong MYO6 signals were detected in the cuticular plate and the cytoplasm in both IHCs and OHCs from the apical, middle, and basal areas of the cochleae of DT-treated wild-type mice (Figs 2b and S2), suggesting that DT administration does not affect the hair cells of wild-type mice. In contrast, the signal disappeared from the OHC areas of the cochleae of DT-treated *Prestin*-*hDTR* mice (Figs 2b and S2). To determine the time at which OHC loss had occurred, we counted the number of MYO6-positive OHCs from the apical, middle, and basal areas of the cochlea 0, 3, 4, and 7 days after administration of DT (Fig. 2a). Although there was no distinct loss of OHCs in *Prestin*-*hDTR* mice several hours (day 0) after administration of DT, we detected approximately 21, 23, and 40% OHC loss at the apical, middle, and basal turns, respectively, on day 3 (Fig. 2c), suggesting that OHC depletion occurred starting from the basal turn of the cochlea. After 4 days, most of the OHC loss had occurred, with 0 to 6% of the cells remaining in the apical, middle, and basal turns of DT-treated *Prestin*-*hDTR* mice (Fig. 2b). The rates of OHC loss in DT-treated *Prestin*-*hDTR* mice were similar after 7 days.

Moreover, the abnormal phenotypes of OHCs associated with OHC-TRECK were confirmed by histopathological analysis by means of light microscopy (LM) and scanning electron microscopy (SEM). The three OHC nuclei and the single IHC nucleus could be observed by LM in untreated *Prestin*-*hDTR* mice. In contrast, the DT-treated *Prestin*-*hDTR* mice had no OHC nuclei and exhibited severe degeneration of the tunnel of Corti (Fig. 2d). According to LM analysis, there were no obvious differences in the phenotypes of spiral ganglion cells (SGCs) and the stria vascularis between the untreated and DT-treated *Prestin*-*hDTR* mice (Fig. 2d). SEM analysis revealed the phenotypes of the apical surfaces of OHCs in DT-treated *Prestin*-*hDTR* mice. V-shaped stereocilia, which is the typical morphology for OHC stereocilia, were rarely detected in DT-treated *Prestin*-*hDTR* mice (Fig. 2e). The surfaces of most OHCs showed loss of stereocilia and the typical scar formed by apical expansion of neighboring supporting cells to close the lesions resulting from OHC loss^12,14^.

**Figure 3:**
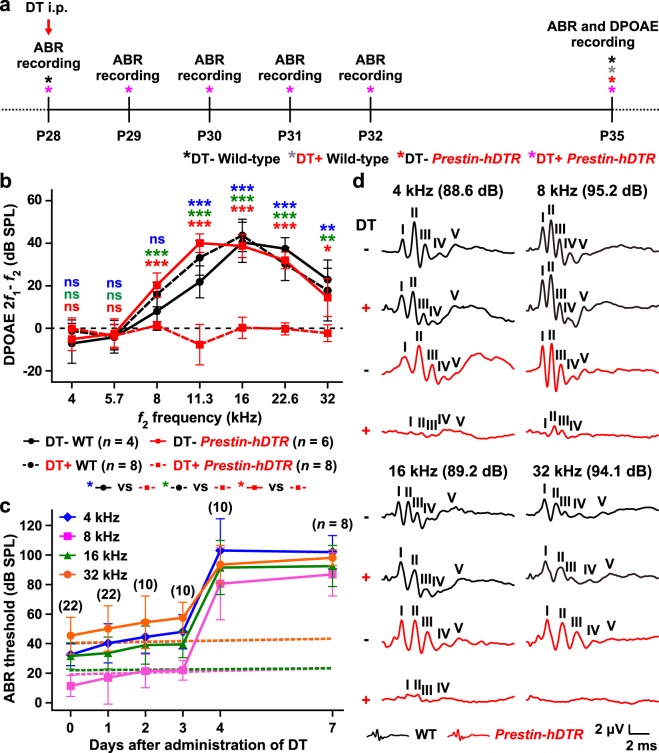
Onset of severe hearing loss in *Prestin*-*hDTR* mice after OHC-TRECK. (**a**) Timelines of experiments and DT administration for recording of the DPOAEs and ABRs. Asterisks indicate the days for recording of DPOAEs and ABRs. (**b**) DPOAE levels in *Prestin*-*hDTR* mice after administration of DT. The means (black circles and red squares) and SDs (error bars) of DPOAE output levels at 2*f*_1_ − *f*_2_ against the *f*_2_ frequencies (4, 5.7, 8, 11.3, 16, 22.6, and 32 kHz) for 50 dB SPL are shown for untreated (DT−) and DT-treated (DT+) wild-type (WT) and DT ± *Prestin*-*hDTR* mice. Asterisks indicate statistical significances: *P < 0.05, **P < 0.01, and ***P < 0.001 (one-way ANOVA with Tukey’s multiple comparisons test). ns, no significant difference. (**c**) Change in ABR thresholds in *Prestin*-*hDTR* mice after administration of DT. The means (symbols) and SDs (error bars) of ABR thresholds for sound stimuli at 4, 8, 16, and 32 kHz are shown for *Prestin*-*hDTR* mice at 0, 1, 2, 3, 4, and 7 days after administration of DT. The number of mice examined is shown. The blue, pink, green, and orange horizontal dotted lines indicate the means of the ABR thresholds for sound stimuli at 4, 8, 16, and 32 kHz, respectively, in WT mice at P28 (*n* = 18) and P35 (*n* = 9). (**d**) Representable ABR waveforms from DT− and DT+ groups of WT and *Prestin*-*hDTR* mice at P35. The waveforms of mice represent the ABRs to tone pip stimuli at the indicated sound pressure level (dB SPL) at 4, 8, 16, and 32 kHz. The locations of ABR peaks I-V are indicated with ranges (μV) of the negative wave apex and latency (ms).

Next, we recorded the distortion product otoacoustic emission (DPOAE) and auditory brainstem response (ABR) in wild-type and DT-treated *Prestin*-*hDTR* mice to confirm the expected decrease or loss of hearing ability by OHC-TRECK (Fig. 3a). We also evaluated the hearing abilities of DT-treated wild-type and untreated *Prestin*-*hDTR* mice to determine whether integration of the transgene and administration of DT had any effect on hearing. Loss of DPOAE, which is a measure of OHC function^30,31^, was detected in the DT-treated *Prestin*-*hDTR* mice (Fig. 3b). Although determination of DPOAE amplitudes was difficult at low frequencies (4, 5.7, and 8 kHz), the DPOAEs in wild-type, DT-treated wild-type, and untreated mice showed similar levels at all frequencies (Fig. 3b). The ABR thresholds to sound stimuli at 4, 8, 16, and 32 kHz were measured in *Prestin*-*hDTR* mice at several time points after DT administration, as shown in Fig. 3c. The ABR thresholds appeared to slowly increase over 3 days, but the differences were not significant. At day 4, the thresholds increased dramatically, reaching a level that was indicative of severe and profound hearing loss to sound stimuli of all frequencies. Moreover, we confirmed that there were no significant differences in the levels of hearing loss between days 4 and 7 after administration of DT. Figure 3d shows representative ABR waveforms for stimuli at the highest sound pressure level (dB SPL) at each frequency recorded for DT-treated and untreated wild-type and *Prestin*-*hDTR* mice. Similar and clear ABR wave I-V amplitudes were detected at all frequencies for the wild-type, DT-treated wild-type, and untreated *Prestin*-*hDTR* mice. In contrast, the ABR amplitudes for DT-treated *Prestin*-*hDTR* mice were remarkably decreased at all frequencies.

### Evaluation of selective OHC depletion by OHC-TRECK

**Figure 4:**
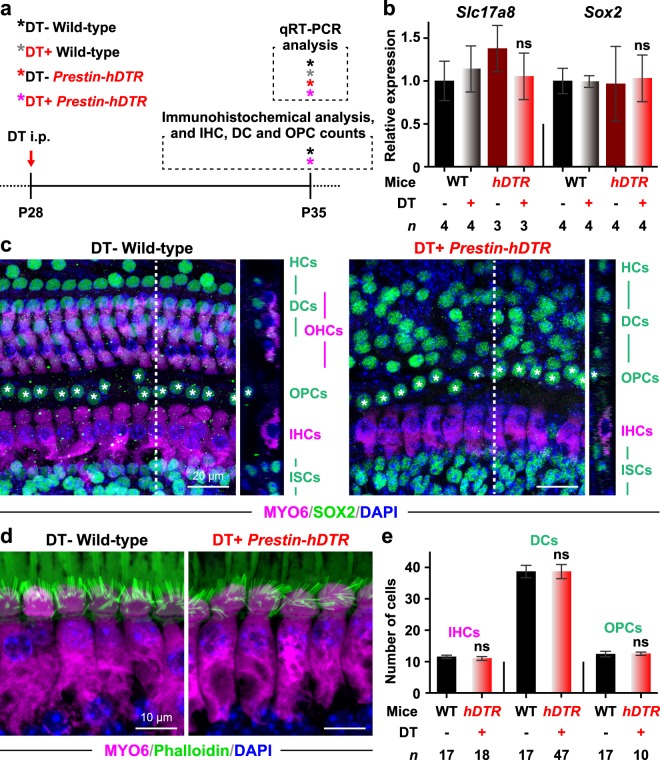
Phenotypes of the IHCs, Deiters’ cells (DCs), and outer pillar cells (OPCs) in *Prestin*-*hDTR* mice after OHC-TRECK. (**a**) Timeline of experiments and DT administration for qRT-PCR analysis, immunohistochemical analysis, and cell counts of the IHCs, DCs, and OPCs. Red arrows and asterisks indicate the days of DT administration and tissue collection for each experiment. (**b**) qRT-PCR analysis of the IHC marker (*Slc17a8*/*Vglut3*) and SC marker (*Sox2*) in cochlear RNA from DT− and DT+ groups of wild-type (WT) and *Prestin*-*hDTR* (*hDTR*) mice. Error bars = SDs. ns, no significance (ANOVA with Tukey’s multiple comparisons test). (**c**) Confocal images of MYO6 (magenta) and SOX2 (green) expression in the OHCs, IHCs, DCs, and OPCs in the middle area of the cochlea in DT− WT and DT+ *Prestin*-*hDTR* mice. The dotted lines in the optical *xy* sectional views in the left panels correspond to the *yz* side views in the right panels. The cochleae were counterstained with DAPI (blue). The OPCs are marked with asterisks. HCs, Hensen’s cells; and ISCs, inner supporting cells (inner pillar, inner phalangeal, and inner border cells). (**d**) Confocal images of the IHCs from the middle area of the cochlea in DT− WT and DT+ *Prestin*-*hDTR* mice. The cochleae of mice were stained with anti-myosin VI (MYO6) antibody (magenta), phalloidin (green), and DAPI (blue). (**e**) Counts of the IHCs, DCs, and OPCs in apical and middle areas in the cochleae of WT and *hDTR* mice. Error bars = SDs. ns, no significance (Student’s t-test).

We next investigated the effects of OHC-TRECK, administration of DT and integration of the transgene in cells neighboring OHCs using qRT-PCR and immunohistochemistry (Fig. 4a). We first quantified the mRNA expression levels of *Slc17a8* (solute carrier family 17, member 8, also known as *Vglut3*) and *Sox2* (SRY-box 2), which are markers of IHCs and supporting cells (SCs), respectively^32,33^. There were no significant differences in the mRNA expression levels of *Vglut3* and *Sox2* in DT-treated *Prestin*-*hDTR* mice (Fig. 4b). Next, we investigated the phenotypes of IHCs and SCs, which include Deiters’ cells (DCs) and outer pillar cells (OPCs), by performing whole-mount staining for phalloidin, anti-MYO6, and anti-SOX2 antibodies (Figs. 4c,d). The morphology of the stereocilia bundles of IHCs and the staining pattern of MYO6 from DT-treated *Prestin*-*hDTR* mice were consistent with those from wild-type mice (Fig. 4d), and there was no change in the number of IHCs in DT-treated *Prestin*-*hDTR* mice (Fig. 4e). Although the organization of SOX2-positive nuclei was slightly disrupted in DT-treated *Prestin*-*hDTR* mice (Fig. 4c), the number of DCs, which intercalated with the three rows of OHCs in their apical phalangeal processes^33,34^, after DT treatment of *Prestin*-*hDTR* mice was similar to that of wild-type mice (Fig. 4e). There were also no significant changes in the number of SOX2-positive nuclei from the OPCs, which are in direct contact with the first row of OHCs^33^, by OHC-TRECK (Fig. 4e).

**Figure 5:**
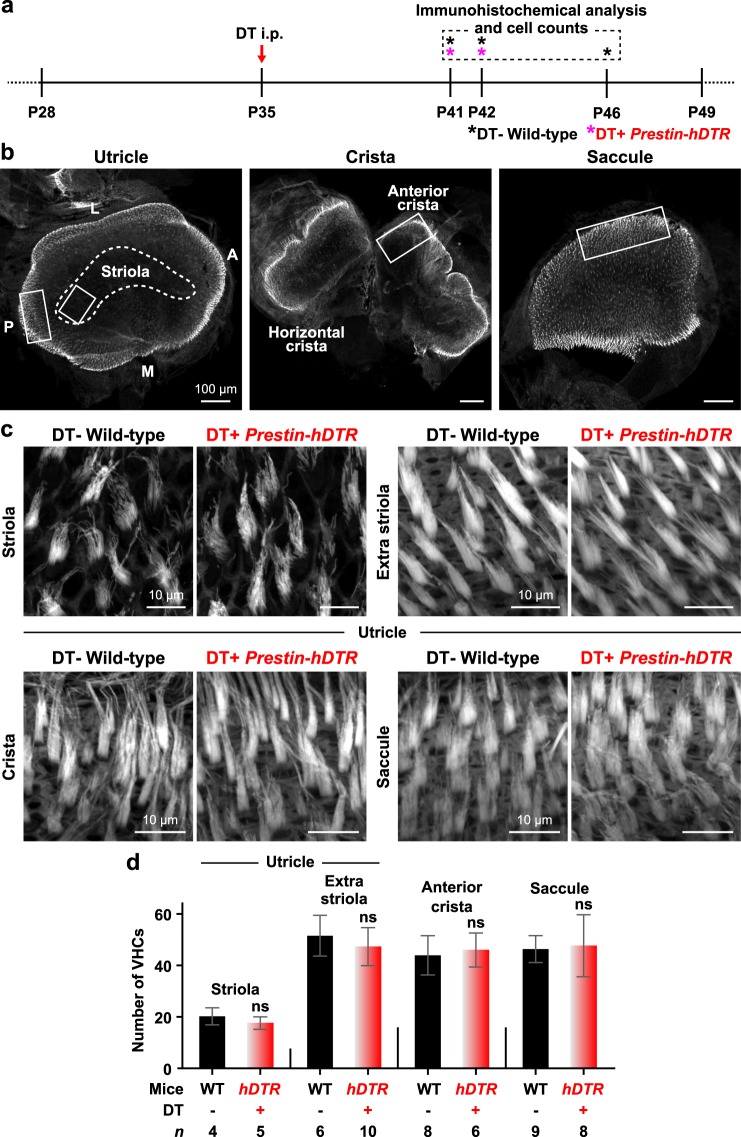
Phenotypes of vestibular hair cells (VHCs) in *Prestin*-*hDTR* mice after OHC-TRECK. (**a**) Timelines of experiments and DT administration for immunohistochemical analysis and VHC counts. Asterisks indicate the days of tissue collection. (**b**) Confocal images of the utricles, cristae, and saccules in mice. The white dotted line is an approximate outline of the striolar region in the utricle. The VHCs were counted within the white boxed areas. A, anterior; L, lateral; M, medial; and P, posterior. (**c**) Confocal images showing the VHCs from the striolar and extrastriolar regions of the utricles, cristae, and saccules of wild-type (WT) and DT-treated (DT+) *Prestin*-*hDTR* mice visualized by phalloidin staining. (**d**) VHC counts in the striolar and extrastriolar regions of the utricles, anterior cristae, and saccules of WT and DT+ *Prestin*-*hDTR* (*hDTR*) mice. Error bars = SDs. ns, no significant difference (Student’s t-test).

**Figure 6:**
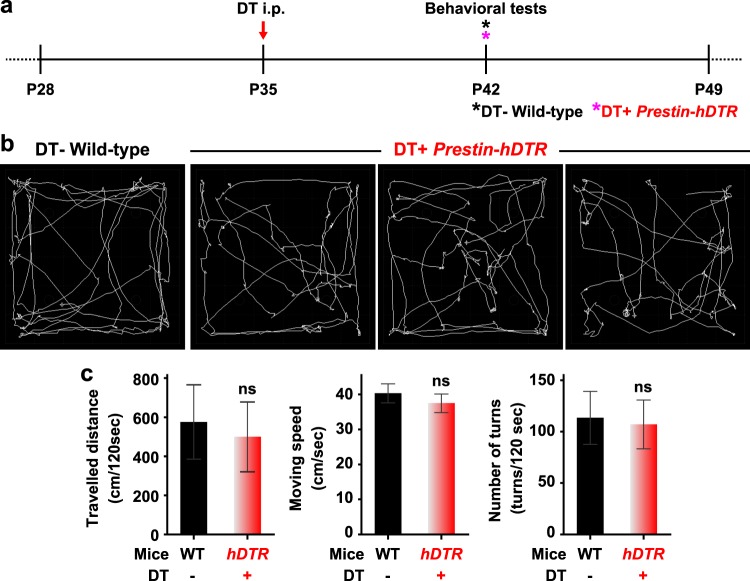
Vestibular functions in *Prestin*-*hDTR* mice after OHC-TRECK. (**a**) Timeline of experiments and DT administration for open-field behavioral tests. Asterisks indicate the days of testing. (**b**) Examples of open-field pathway traces (white lines) for wild-type (WT) and DT-treated (DT+) *Prestin*-*hDTR* mice. (**c**) Quantitation of the average traveled distance, movement speed, and number of turns in 120 sec from 44 individuals of each WT and DT+ *Prestin*-*hDTR* (*hDTR*) mouse. Error bars = SDs. ns, no significant difference (Student’s t-test).

Moreover, we investigated the effect of OHC-TRECK on VHCs in the utricle, crista, and saccule (Fig. 5a,b). The morphologies of the VHC stereocilia and the number of VHCs from DT-treated *Prestin*-*hDTR* mice were the same as those from wild-type mice in the three vestibular tissues (Fig. 5c,d). We also investigated the vestibular functions of DT-treated *Prestin*-*hDTR* mice (Fig. 6a) by open-field behavior tests. The tests confirmed that the behaviors of DT-treated *Prestin*-*hDTR* mice were normal, and the mice did not exhibit hyperactivity or circling behavior, which were observed in mice with vestibular dysfunction (Fig. 6b,c).

### Changes in gene expression profiles in the cochlea by OHC-TRECK

We next performed differential gene expression analysis of cochlear RNAs isolated from the wild-type, DT-treated wild-type, *Prestin*-*hDTR*, and DT-treated *Prestin*-*hDTR* mice using a microarray to determine the changes in gene expression in OHCs after OHC-TRECK. We first investigated the effects of transgene integration on gene expression by comparing differentially expressed genes (DEGs) between wild-type and *Prestin*-*hDTR* mice. We found that 92 and 232 probes were up- and downregulated, respectively, with a ≥2-fold change (Supplementary Fig. S3a,c). We also determined the DEGs between wild-type and DT-treated wild-type mice to investigate the effect of DT treatment on gene expression. This analysis revealed that 238 probes were differentially expressed; 173 were upregulated and 65 were downregulated by DT administration (Supplementary Fig. S3b,d). Thus, these results indicated that transgene integration and DT treatment may influence gene expression profiles. Moreover, we identified the common up- and downregulated probes, which could lead to a bias in data analysis due to the effects of the transgene and DT administration, by pairwise comparisons between the “wild-type vs *Prestin*-*hDTR*” and “wild-type vs DT-treated *Prestin*-*hDTR*” groups (Supplementary Fig. S3a,c) and between the “wild-type vs DT-treated wild-type” and “wild-type vs DT-treated *Prestin*-*hDTR*” groups (Supplementary Fig. S3b,d). We found 113 common up- and downregulated probes (7 in Supplementary Fig. S3a; 24 in Supplementary Fig. S3b; 50 in Supplementary Fig. S3c; and 32 in Supplementary Fig. S3d), and these probes were removed from further analysis in this study.

**Figure 7:**
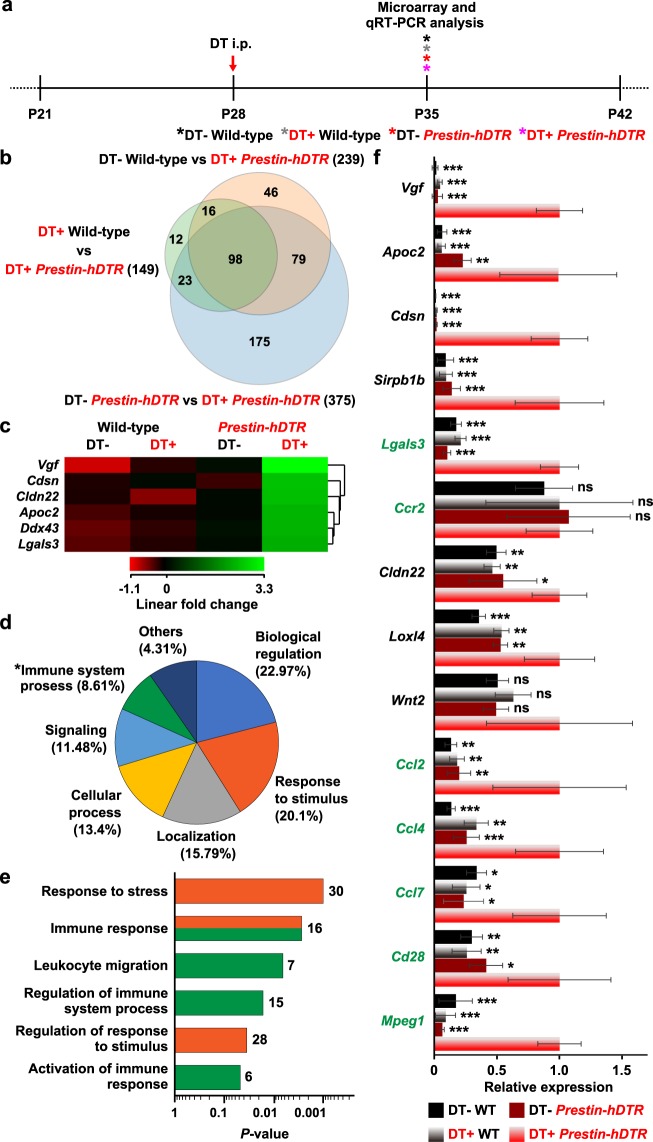
Characterization of the upregulated genes in cochlear RNA by OHC-TRECK. (**a**) Timelines of experiments and DT administration for microarray and qRT-PCR analysis. Asterisks indicate the days of tissue collection for RNA extraction. (**b**) Venn diagram illustrating the common and unique upregulated genes (fold change ≥2) in cochlear RNA across untreated (DT−) wild-type (WT) vs DT-treated (DT+) *Prestin*-*hDTR* mice, DT− *Prestin*-*hDTR* vs DT+ *Prestin*-*hDTR* mice, and DT+ WT vs DT+ *Prestin*-*hDTR* mice. (**c**) Gene expression profiles of the top 1 (*Vgf*), 2 (*Ddx43*), 3 (*Apoc2*), 4 (*Cdsn*), 6 (*Lgals3*), and 9 (*Cldn22*) highly upregulated genes by OHC-TRECK. A cluster cropped from the heat map (Supplementary Fig. S4) of 98 common probes (94 genes) (**b**). (**d**,**e**) Gene ontology (GO) analysis of 98 commonly upregulated genes by OHC-TRECK. A pie chart with 7 subdivisions shows GO terms for the “biological process” category (**d**). *P = 0.0437. The horizontal bar chart shows the enriched GO terms within “response to stimulus” (orange bars) and “immune system process” (green bars) with statistical significance (*P* < 0.05). The number of genes within each category are indicated on the right side of each bar (**e**). (**f**) Validation of the microarray data. The expression levels of nine genes (*Vgf*, *Apoc2*, *Cdsn*, *Sirpb1b*, *Lgals3*, *Ccr2*, *Cldn22*, *Loxl4*, and *Wnt2*) among the top 11 highly upregulated genes and five immune-related genes (*Ccl2*, *Ccl4*, *Ccl7*, *Cd28*, and *Mpeg1*) were validated in the cochlear RNA of DT ± WT and *Prestin*-*hDTR* (*P*-*hDTR*) mice. The seven immune-related genes are highlighted in green. Error bars = SDs. Asterisks indicate statistical significance (*P < 0.05, **P < 0.01, and ***P < 0.001: one-way ANOVA with Tukey’s multiple comparisons test) compared with DT+ *P*-*hDTR* mice. ns, no significant difference.

Gene expression analysis identified 449 upregulated probes (fold change cutoff of 2) (Fig. 7b). Within the probe sets, 98 probes (94 genes) overlapped in pairwise comparisons among the “wild-type vs DT-treated *Prestin*-*hDTR*”, “DT-treated wild-type vs DT-treated *Prestin*-*hDTR*”, and “*Prestin*-*hDTR* vs DT-treated *Prestin*-*hDTR*” groups (Fig. 7b and Supplementary Table S1), and these probes were further analyzed as candidates of OHC-TRECK-specific upregulated genes. Figure 7c highlights a cluster that contains 6 of the top 10 differentially upregulated genes (Supplementary Fig. S4) from the 98 probe sets. The most highly upregulated gene (fold change of +18.38) was *Vgf* (VGF nerve growth factor inducible), which is a neuronal polypeptide that is known to be overexpressed by nerve injury and endoplasmic reticulum stress-induced cell death as a neuroprotective effect^35,36^. Gene ontology (GO) analysis of the 94 genes revealed 79 significant GO terms of the “biological process” category (P < 0.05) (Supplementary Table S2). Within the category of “biological process”, the enriched GO terms were “biological regulation” (22.97%) and “response to stimulus” (20.1%) (Fig. 7d). Moreover, the association with the GO term “immune system process” was statistically significant (*P* = 0.0437). Among the broader GO terms, there was a significant association with “response to stress”, “immune response”, “leukocyte migration”, “regulation of immune system process”, “regulation of response to stimulus”, and “activation of immune response” (Fig. 7e). Moreover, we observed upregulation (fold change of +4.78) of *Wnt2* (wingless-type MMTV integration site family, member 2), the expression of which had previously been reported to be significantly increased 3 days after DT treatment in RNA from *iDTR*;*Gfi1*-Cre mice^37^ (Supplementary Fig. S4 and Supplementary Table S1). The OHC-TRECK-specific upregulated genes in the microarray analysis were validated using qRT-PCR analysis. This analysis confirmed that eight genes among the top 11 highly upregulated genes in the microarray analysis were significantly upregulated in the cochlear RNA from DT-treated *Prestin*-*hDTR* mice, although the expression of *Ddx43* (DEAD box polypeptide 43) and *Gm10433* (uncharacterized noncoding RNA gene) was undetectable by qRT-PCR, and the expression levels of *Ccr2* and *Wnt2* were not significantly upregulated in DT-treated *Prestin*-*hDTR* mice (Fig. 7f). We also validated the expression levels of five immune genes, which were predicted to be highly expressed in the cochlea from microarray data. The expression levels were significantly upregulated in DT-treated *Prestin*-*hDTR* mice (Fig. 7f).

**Figure 8:**
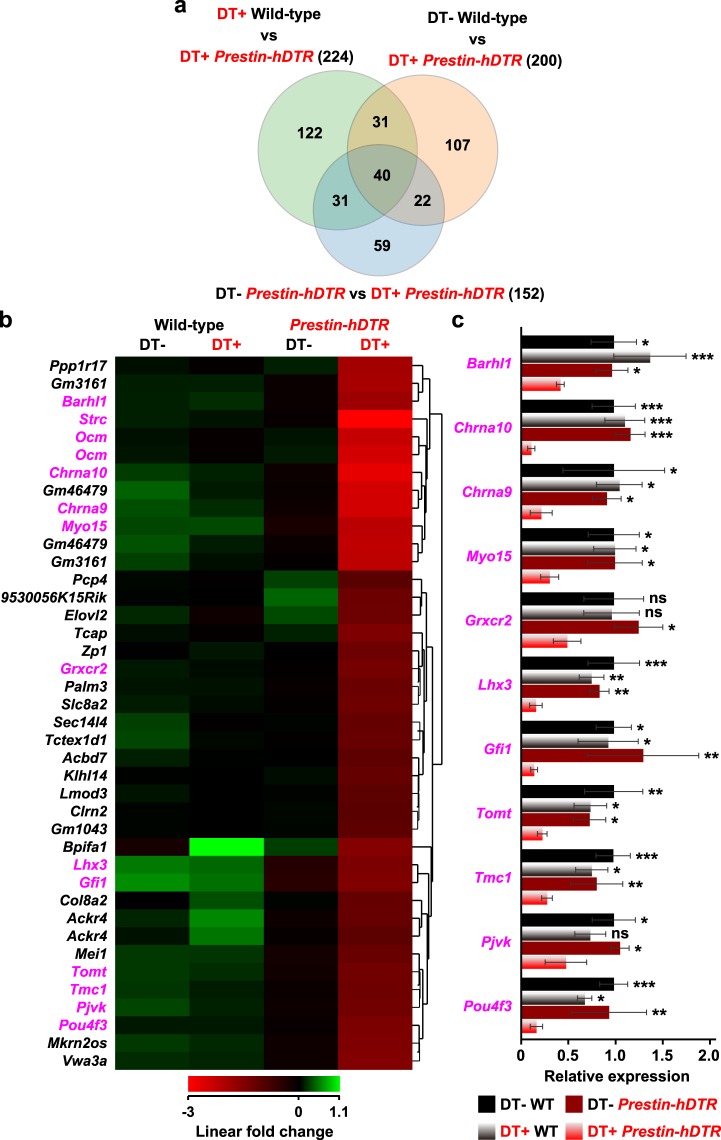
Characterization of the downregulated genes in cochlear RNA by OHC-TRECK. (**a**) Venn diagram illustrating the common and unique downregulated genes (fold change ≤−2) in cochlear RNA across untreated (DT−) wild-type (WT) vs DT-treated (DT+) *Prestin*-*hDTR*, DT− *Prestin*-*hDTR* vs DT+ *Prestin*-*hDTR*, and DT+ WT vs DT+ *Prestin*-*hDTR* mice. (**b**) Heat map showing the expression profiles of 40 common probes (**a**), including 36 genes downregulated in DT+ *Prestin*-*hDTR* mice. The genes responsible for hearing loss in humans and mice are highlighted in magenta. (**c**) Validation of the microarray data. The genes responsible for hearing loss in humans and mice were sued to validate the expression levels in the cochlear RNA of DT ± WT and *Prestin*-*hDTR* (*P*-*hDTR*) mice. Error bars = SDs. Asterisks indicate statistical significance (*P < 0.05, **P < 0.01, and ***P < 0.001: one-way ANOVA with Tukey’s multiple comparisons test) compared with DT− WT mice. ns, no significant difference.

We confirmed that 40 probes (36 genes) were differentially downregulated more than two-fold by pairwise comparison among the “wild-type vs DT-treated *Prestin*-*hDTR*”, “DT-treated wild-type vs DT-treated *Prestin*-*hDTR*”, and “*Prestin*-*hDTR* vs DT-treated *Prestin*-*hDTR*” groups (Fig. 8a,b and Supplementary Table S3). GO analysis confirmed that 15 of the 36 genes were significantly associated with hearing-related GO terms (Supplementary Table S4). Moreover, mutations and/or deletions of 13 genes, namely, *Strc*, *Chrna10* (cholinergic receptor, nicotinic, alpha polypeptide 10), *Chrna9* (cholinergic receptor, nicotinic, alpha polypeptide 9), *Ocm*, *Myo15* (myosin XV), *Gfi1* (growth factor independent 1), *Lhx3* (LIM homeobox protein 3), *Barhl1* (BarH-like 1 (Drosophila)), *Pjvk* (pejvakin), *Tomt* (transmembrane O-methyltransferase), *Pou4f3* (POU domain, class 4, transcription factor 3), *Tmc1* (transmembrane channel-like gene family 1), and *Grxcr2* (glutaredoxin, cysteine rich 2), cause hearing loss in humans and mice (Fig. 8b)^26–28,38–50^. The ten most downregulated genes (fold change of −8.52 to −3.88) were *Strc*, *Chrna10*, *Gm46479* (uncharacterized noncoding RNA), *Chrna9*, *Ocm*, *Gm3161* (predicted gene), *Myo15*, *Gfi1*, *Ppp1r17* (protein phosphatase 1, regulatory subunit 17), and *Lhx3* (LIM homeobox protein 3) (Supplementary Table S3). High and/or specific expression of *Strc*, *Chrna10*, *Chrna9*, and *Ocm* in OHCs has been demonstrated previously^26–28,51,52^. We performed qRT-PCR analysis to validate the downregulation of the genes responsible for hearing loss in humans and mice. The analysis confirmed significant downregulation of these genes in the cochlear RNA from DT-treated *Prestin*-*hDTR* mice (Figs 1e and 8c).

### OHC-TRECK in neonatal mice

**Figure 9:**
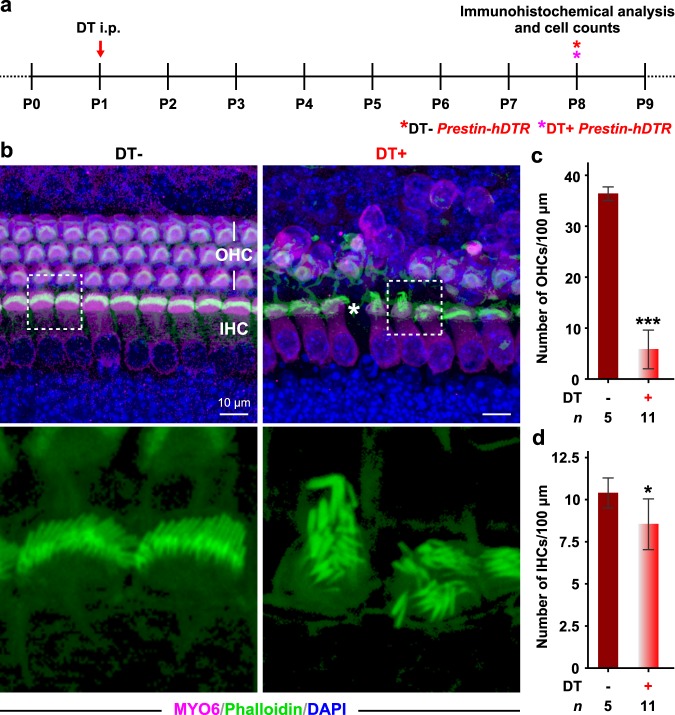
Partial depletion of OHCs and damage of IHCs in neonatal *Prestin*-*hDTR* mice after administration of DT. (**a**) Experimental design for immunohistochemical analysis and OHC counts. Asterisks indicate the days of tissue collection. (**b**) Confocal images showing the OHCs and IHCs of untreated (DT−) and DT-treated (DT+) *Prestin*-*hDTR* mice visualized by anti-MYO6 antibody (magenta), phalloidin (green), and DAPI (blue). High-magnification images of the stereocilia bundles indicated in dotted boxes in the top panels are shown in bottom panels. The asterisk indicates the IHC loss. (**c**,**d**) OHC (**c**) and IHC (**d**) counts in the middle areas in the cochleae of DT− and DT+ *Prestin*-*hDTR* mice. Error bars = SDs. Asterisks indicate statistical significance: *P < 0.05 and ***P < 0.001 (Student’s t-test).

Next, we investigated the phenotypes associated with OHC-TRECK in neonatal mice (P1) using immunohistochemical analysis (Fig. 9a). The number of OHCs in neonatal mice was clearly decreased in *Prestin*-*hDTR* mice 7 days after administration of DT (Fig. 9b,c). However, a few OHCs survived in the cochleae of DT-treated juvenile *Prestin*-*hDTR* mice at P8 compared with those of adult stages. Moreover, obvious damage was observed in the IHCs of DT-treated *Prestin*-*hDTR* mice. The number of IHCs decreased significantly in DT-treated *Prestin*-*hDTR* mice (Fig. 9b,d). Abnormal stereocilia bundles were observed in many IHCs from DT-treated *Prestin*-*hDTR* mice.

## Discussion

Several cellular knockout methods for hair cells and SCs using DT have been established and are widely utilized in studies of hair cell regeneration^37,53,54^, the innate immune system in cochlear cells^55^, SGC survival after hair cell death^56^, and the relationship between vocalization and auditory experience^57^. In this study, we established *Prestin*-*hDTR* mice, which allow *in vivo* depletion of almost all OHCs approximately 4 days after i.p. administration of DT (Figs 1d, 2b,c and S2). Although the same effect on OHCs can be achieved by tamoxifen-mediated induction in *Prestin*^*CreERT2*+/−^;*Rosa26*^*DTA*/−^ mice^58^, *Prestin*-*hDTR* mice exhibit OHC depletion by simple DT administration, which will prove useful for understanding the consequences of OHC death.

Moreover, our results suggested that the OHC-TRECK method exhibits selective depletion without any damage to IHCs, SCs (DCs and OPCs), SGCs, the stria vascularis, and VHCs (Figs 2d, 4 and 5) 7 days after administration of DT. This selective depletion is very important because the effects of specifically killing OHCs on other cell types within the cochlea will be investigated by morphological and electrophysiological phenotypic analysis and gene expression analysis in short- and long-term studies. In addition, we expect that OHC-TRECK will be very useful for the comparison of models with noise- or ototoxic drug-induced HC death and to determine how these insults impact or damage other cell types.

We also investigated the effect of transgene integration and DT treatment on the auditory phenotypes and gene expression of DT-treated and untreated wild-type and *Prestin*-*hDTR* mice. In particular, the dose (i.p., 50 μg/kg body weight) of DT used for TRECK in this study was extremely high compared with those used in previous studies, although DT administration did not lead to mouse death at this dose^22,23^. Comparison of the cochlear RNAs of untreated *Prestin*-*hDTR* and DT-treated wild-type mice showed that several genes were differentially expressed (Supplementary Fig. S3). Although these results indicate the effects of the transgene and DT in mice, there are no significant GO terms (P < 0.05) associated with specific transgenesis- and DT treatment-related DEGs. Moreover, the hearing levels were normal in untreated *Prestin*-*hDTR* and DT-treated wild-type mice (Fig. 3b,d). Therefore, we predict that the effects of the transgene and DT on auditory phenotypes are extremely weak in adult mice.

Differential gene expression analysis using *Prestin*-*hDTR* mice may provide interesting information related to OHC death. We performed microarray analysis using RNA isolated from cochleae 7 days after DT treatment of *Prestin*-*hDTR* mice as a preliminary experiment and observed the upregulation of several immune-related genes, including cytokine receptors and ligands and CD antigens, which are associated with biological processes such as response to stress, immune response, and leukocyte migration (Figs. 7c–e and S4, Supplementary Tables S1 and S2). Upregulation of immune-related genes probably results from inflammatory/immune responses caused by OHC death. Previous studies have reported the upregulation of genes associated with inflammatory/immune responses by administration of an ototoxic drug^59^, acoustic trauma^60^, and aging^61^. Immune and inflammatory responses are predicted to be essential responses to injury and loss of OHCs; therefore, we suggest that *Prestin*-*hDTR* mice can be used to identify genes associated with these responses. Although we performed cochlear gene expression analysis on day 7 (3 days after the death of almost all OHCs), the use of samples obtained 3 and 4 days after DT administration may be more useful because these are the stages at which OHC death occurs (Fig. 2c).

We also identified specific downregulated genes after selective OHC depletion and predicted that these genes are associated with the maintenance and function of OHCs. As expected, the downregulated genes included several genes responsible for the maintenance and function of OHCs, such as *Strc*, which is essential not only for the formation of the horizontal top connectors of OHC hair bundles but also for the cohesiveness of the mature OHC hair bundles for tip-link turnover^27^; *Chrna9*, and *Chrna10*, which are required for normal synaptic function and integrity of the olivocochlear system in OHC neurons^38,39^; and *Ocm*, which probably plays a role in OHC calcium homeostasis^26^ (Fig. 8b and Supplementary Table S3). Moreover, the expression levels of the other nine deafness-related genes (*Myo15*, *Gfi1*, *Lhx3*, *Barhl1*, *Pjvk*, *Tomt*, *Pou4f3*, *Tmc1*, and *Grxcr2*) were downregulated more than 2-fold in DT-treated *Prestin*-*hDTR* mice. In OHCs from null mutant mice, depletion of *Gfi1*, *Barhl1*, *Pjvk*, *Tomt*, and *Grxcr2* resulted in early-onset degeneration and severe phenotypes compared with IHCs^41,43–45,48^. While the functions of the other downregulated genes in OHCs are not known, analysis of these genes may provide insight into the function and maintenance of OHCs. For example, *Clrn2* (clarin 2), which encodes a transmembrane protein, belongs to the clarin family of genes. Another gene in the same family, *CLRN1* (clarin 1), is a causative gene of Usher syndrome type III^50^. The expression of *Clrn1* is higher in OHCs than in IHCs, and *Clrn1* null mutant mice exhibit disorganization of OHC stereocilia^62^. *Tcap* (titin-cap) is another candidate gene that is responsible for hearing loss. Although *Tcap* is known as a causative gene of muscular dystrophy in humans^63^, the transcript is detected in the otic vesicles of mouse embryos^64^. Moreover, we are interested in *Ppp1r17*, which was one of the ten most downregulated genes. The protein encoded by the *Ppp1r17* gene functions as an inhibitor of the Ser/Thr phosphatases, protein phosphatase 2A (PP2A) and protein phosphatase 1 (PP1)^65^. Liu *et al*. recently reported that aging leads to decreased protein levels of PP1 in the mouse cochlea^66^. Thus, these downregulated genes may contribute to the functions and maintenance of OHCs. In addition, these genes might be candidate causative genes for hearing loss in patients for whom the cause of hearing loss is unknown.

Although we expected that the OHC-TRECK system could be used in OHC studies at early postnatal stages, selective depletion of OHCs was not observed by the administration of DT to neonatal *Prestin*-*hDTR* mice. We observed obvious damage of the IHCs in DT-treated *Prestin*-*hDTR* mice after administration of DT at P1 (Fig. 9). Konishi *et al*. reported that postnatal administration of DT at days 7 and 14 led to decreased hearing levels and degeneration of IHCs, OHCs, and SGCs in wild-type C57BL/6 mice^67^, indicating that administration of DT alone at juvenile stages has several adverse effects on the immature cochlea. Thus, our TRECK system using *Prestin*-*hDTR* mice should be used for OHC studies at adult stages and is currently a limited model for the analysis of OHCs at juvenile stages, although the system might be able to improve the dose and administration method of DT, as several studies have used a similar system for research on hearing at juvenile stages^37,53,55,56^. Another likely explanation for the *Prestin*-*hDTR* model being less effective and selective in the neonatal period is that SLC26A5/prestin expression is low at P1. OHCs were predominantly eliminated by TRECK at P1, but several OHCs survived in almost all the cochleae (Fig. 9). The most prominent increase in prestin expression occurred after P6 in mice and rats^68,69^. Therefore, the efficiencies of OHC depletion could be improved using *Prestin*-*hDTR* mice at later juvenile stages that exhibit raised prestin expression.

## Methods

### Study approval

This study was performed in strict accordance with the recommendations outlined in the Guidelines for Proper Conduct of Animal Experiments by the Science Council of Japan. The protocol was approved by the Institutional Animal Experiment Committee of the Tokyo Metropolitan Institute of Medical Science (permit numbers: 14078, 15045, 16065, 17041, and 18066). All surgery was performed under isoflurane anesthesia, and all efforts were made to minimize suffering.

### Generation of *Prestin*-*hDTR* mice and inducible depletion of OHCs

Consecutive DNA fragments corresponding to the promoter regions of *Sla26a5* were amplified by PCR from the genomic DNA of a C57BL/6J mouse (CLEA Japan, Tokyo, Japan) using the following primer set: 5′-AAG GCT GAC TCA GTG AAG TAG AGT CCA TGC-3′ and 5′-CTC AGA ATC CCC TAG CTC AAG ACA TTC TCG-3′ for the 14.1-kb first site and 5′-AGC TTC CCA TCC CAC CTG TAT TG-3′ and 5′-AGA TCG ATT CAC CAA CAG CAG GAG ACA AGC-3′ for the 13.2-kb second site. The fragments were ligated at the *Sex*AI site. The *Sla26a5* promoter, modified *HB-EGF* cDNA (a kind gift from Kenji Kono, Nara Institute of Science and Technology)^25^ encoding the I117V/L148V mutant, rabbit *β-globin* and SV40 pA^+^ were ligated and cloned into the pBluescript II SK(+) vector (Agilent Technologies, Santa Clara, CA, USA). The 28.3-kb *Not*I-*Sal*I fragment was excised and purified using the QIAquick Gel Extraction Kit (QIAGEN, Valencia, CA, USA) and the Wizard DNA Clean-Up System (Promega, Madison, WI, USA). *Prestin*-*hDTR* mice were generated by microinjection of the DNA construct into pronucleus-stage oocytes from C57BL/6J mice. Genomic DNA was purified from tail biopsies using the Wizard Genomic DNA Purification Kit (Promega). PCR genotyping was performed for genomic DNA using the following primer set: 5′- ATA TCG ATT CGA AAG TGA CTG GTG CCT CGC-3′ and 5′-AGA CAG ACA GAT GAC AGC ACC ACA G-3′ for the *HB-EGF* cDNA. The mice were i.p. administered DT at a dose of 50 μg/kg body weight.

### qRT-PCR

Total RNA was isolated from the cochleae of wild-type, DT-treated wild-type, *Prestin*-*hDTR* and DT-treated *Prestin*-*hDTR* mice at P35 (Figs 1c, 4a and 7a) using the PureLink RNA Mini Kit (Thermo Fisher Scientific, Grand Island, NY, USA) according to the manufacturer’s instructions. qRT-PCR was performed using TB Green Premix Ex Taq II (Tli RNaseH Plus) (Takara Bio Inc., Kyoto, Japan) and primer sets for 29 target genes, with *Gapdh* (glyceraldehyde-3-phosphate dehydrogenase) as an internal control (Supplementary Table S5). qRT-PCR primers were designed for separate exons, except those for *Sox2* and *Ccr2*, which are intron-less genes. The products were analyzed on a LightCycler 480 System II instrument (Roche Molecular Systems, Inc., Pleasanton, CA, USA). The signal values were normalized to the median *Gapdh* signals, and the geometric mean values of the target signals were calculated in triplicate. The expression levels of the genes in untreated wild-type (Figs. 1e, 4b and 8c) and DT-treated *Prestin-hDTR* (Fig. 7f) mice were assigned an arbitrary value of 1 for comparison.

### Immunohistochemistry

The inner ears were removed from the heads of mice of several ages (Figs 1c, 2a, 4a, 5a and 9a) and fixed with 4% paraformaldehyde (PFA). The cochlear and vestibular tissues were dissected from inner ears that were decalcified in 5% EDTA/PBS at 4 °C for 2 days and from non-decalcified inner ears. The tissues were permeabilized in 0.25% Triton X-100 in PBS for 15–30 min and then subjected to three 5-min washes in PBS. After the samples were washed in PBS, nonspecific binding sites were blocked with 0.5% Blocking Reagent (Roche Molecular Biochemicals, Indianapolis, IN, USA) for 1 hour at RT. Samples were incubated with primary antibody diluted in Can Get Signal Immunostain Solution A (TOYOBO, Osaka, Japan) overnight at 4 °C. The following primary antibodies were used: rabbit polyclonal anti-SLC26A5/prestin (Santa Cruz Biotechnology, Dallas, TX, USA; sc-30163, 2 μg/ml), rabbit polyclonal anti-MYO6 (Proteus Biosciences Inc, Ramona, CA, USA; 25–6791, 5 μg/ml), and goat polyclonal anti-SOX2 (Santa Cruz; sc-17320, 2 μg/ml) antibodies. The samples were then washed three times for 5 min in PBS, and an Alexa Fluor 594-conjugated secondary antibody (Thermo Fisher Scientific) and Alexa Fluor 488-conjugated phalloidin (Thermo Fisher Scientific) were diluted to 20 μg/ml and 4 units/ml, respectively, in Can Get Signal Immunostain Solution B (TOYOBO) for 1 hour at RT. Finally, the samples were washed three times for 5 min in PBS and then mounted onto glass slides using ProLong Gold antifade reagent (Thermo Fisher Scientific). Fluorescence images were obtained using a Zeiss LSM 710 confocal microscope (Carl Zeiss, Jena, Germany), and images were processed using ZEN2009 software (Carl Zeiss). The MYO6-positive OHCs and IHCs, and SOX2-positive DCs and OPCs per 100 μm were counted from the apical (1.2–1.5 mm from the apex), middle (2.1–2.4 mm from the apex), and basal (2.0–3.4 mm from the base) areas in the cochleae. The VHCs visualized by phalloidin were counted from the 2,500-μm^2^ area within white boxes in the utricle, crista, and saccule as shown in Fig. 5b.

### Histological analysis

For preparation of paraffin-embedded cochlear sections, isoflurane-anesthetized mice were perfused through the heart with a buffer containing 4% PFA. The inner ears of *Prestin*-*hDTR* and DT-treated *Prestin*-*hDTR* mice at P42 (Fig. 2a) were dissected, fixed with 4% PFA overnight, and then decalcified in 5% EDTA/PBS at 4 °C. After decalcification for 14 days, tissues were dehydrated in an ethanol series, cleared in xylene, embedded in paraffin, sectioned (5 μm), and stained with hematoxylin.

SEM of the apical surfaces of OHCs in the ears of untreated and DT-treated *Prestin*-*hDTR* mice at P49 (Fig. 2a) was performed as previously described^70^ using a Hitachi S-4800 field emission SEM instrument at an accelerating voltage of 10 kV.

### Hearing tests

The hearing ability of the mice was evaluated by recording the DPOAE and ABR. To perform these hearing tests, mice were anesthetized by i.p. injection of a medetomidine-midazolam-butorphanol mixture (0.75 mg/kg medetomidine, 4 mg/kg midazolam, and 5 mg/kg butorphanol). After the experiment, mice were administered the alpha-2 adrenergic antagonist atipamezole (0.75 mg/kg).

DPOAEs were recorded from each ear of the wild-type, DT-treated wild-type, *Prestin*-*hDTR*, and DT-treated *Prestin*-*hDTR* mice at P35 (Fig. 3a) using the ER10X Extended Bandwidth Research Probe System (Etymotic Research, Elk Grove, IL, USA) and EMAV Plus software (version 3.32) (Etymotic Research). For recording DPOAEs, a small ear plug containing a low-distortion probe microphone (Etymotic Research, ER10X-P) and two probe tubes was set in the outer ear canal. Two test sounds were separately introduced into the ear through two tubes. The primary tone stimulus levels L_1_ and L_2_ were adjusted as L_1_ (65 dB SPL) - L_2_ (55 dB SPL) = 10 dB SPL. The frequency ratio *f*_2_/*f*_1_ was fixed at 1.20, with *f*_2_ = 32, 22.6, 16, 11.3, 8, 5.7, and 4 kHz. The cubic distortion product of 2*f*_1_ − *f*_2_ was measured as DPOAE.

ABRs evoked by tone pip stimuli for each pressure level at 4, 8, 16, and 32 kHz were recorded from each ear of the wild-type, DT-treated wild-type, *Prestin*-*hDTR*, and DT-treated *Prestin*-*hDTR* mice at various ages, as shown in Fig. 3a, using the ABR Workstation (TDT System III, Alachua, FL, USA) as previously described^70^. ABR thresholds were obtained for each stimulus by reducing the SPL first in 10-dB steps and then fluctuating the SPL in 5-dB steps to identify the lowest level at which an ABR pattern could be recognized.

### Behavioral test

The wild-type and DT-treated *Prestin*-*hDTR* mice at P42 (Fig. 6a) were placed in a 50 cm × 40 cm × 50 cm (W × H × L) open field to quantify behaviors as previously described^70^. Data on the total traveled distance (cm/120 sec), average moving speed (cm/sec) and number of turns (times/120 sec) were collected and analyzed using CompACT VAS software ver. 3.1 (Muromachi Kikai, Tokyo, Japan).

### Microarray analysis

The cochleae (*n* = 8 per mouse) of wild-type, DT-treated wild-type, *Prestin*-*hDTR*, and DT-treated *Prestin*-*hDTR* mice at P35 (Fig. 7a) were used for microarray analysis. Total RNA samples from each mouse were prepared as mentioned above, and the qualities were assessed with a Bioanalyzer 2100 (Agilent Technologies). Two hundred nanograms of total RNA was amplified and labeled using the Agilent Low Input Quick Amp Labeling Kit (Agilent Technologies) according to the manufacturer’s protocol. A total of 1.65 µg of Cy3-labeled cRNA was fragmented using the protocol for the Agilent Gene Expression Hybridization Kit. Hybridizations were performed for 17 hours at 65 °C and 10 rpm in a rotating hybridization oven. Slides were scanned with the SureScan microarray scanner (Agilent Technologies) using a scan resolution of 5 µm and the green dye channel with the PMT set to 100%. Data were obtained using Agilent Feature Extraction software (v11.5.1.1) with default settings for all parameters. Data mining was performed using GeneSpring GX ver. 14.9 software (Agilent Technologies). Normalization of the raw data was performed using the percentile shift (75th percentile) followed by baseline transformation to the medians of all samples. The DEGs were screened for a 2-fold change between samples. To interpret the results of transcriptome analysis, GO analysis using GeneSpring GX was conducted for DEGs between the DT-treated *Prestin*-*hDTR* mice and other samples (untreated wild-type, DT-treated wild-type, and untreated *Prestin*-*hDTR* mice). Microarray data have been deposited in the Gene Expression Omnibus (GEO) database at NCBI (https://www.ncbi.nlm.nih.gov/geo/) under accession number GSE121442.

### Statistical analysis

All results are presented as the mean ± standard deviation (SD). The differences in mRNA expression levels (Fig. 1e), number of cells (IHCs, DCs, OPCs, and VHCs in adult mice, and OHCs and IHCs in neonatal mice) (Figs 4e, 5d and  9c,d) and behavioral data (Fig. 6c) were analyzed using Student’s t-test. The number of OHCs at the adult stage was statistically analyzed by a two-way ANOVA with a Bonferroni post-hoc, including the data for all the days for both DT-treated wild-type and *Prestin*-*hDTR* mice. Differences in DPOAE levels (Fig. 3b) and mRNA expression levels (Figs 4b, 7f and 8c) were analyzed by a one-way ANOVA with the Tukey post hoc multiple comparison test. GraphPad Prism6 (GraphPad, San Diego, CA, USA) was used to calculate the column statistics and P values.

## Supplementary information

Supplementary Information

## Data Availability

All data obtained or analyzed during this study are included in this manuscript. Raw data are available from the corresponding author upon reasonable request.
