# Supplementary material for: OHC-TRECK: A Novel System Using a Mouse Model for Investigation of the Molecular Mechanisms Associated with Outer Hair Cell Death in the Inner Ear
Source: Sci Rep. 2019 Mar 27;9:5285. doi: 10.1038/s41598-019-41711-2 (PMC6437180; doi:10.1038/s41598-019-41711-2)
Supplement: Supplementary file 1 — Supplementary Information [file 41598_2019_41711_MOESM1_ESM.pdf]

OHC-TRECK: A Novel System Using a Mouse Model for Investigation of the Molecular Mechanisms Associated with Outer Hair Cell Death in the Inner Ear

Kunie Matsuoka[1], Kenta Wada[1,2], Yuki Miyasaka[1,3], Shumpei P. Yasuda[1], Yuta Seki[1], Yasumasa Nishito[4], Hiromichi Yonekawa[5], Choji Taya[5], Hiroshi Shitara[5] & Yoshiaki Kikkawa[1]

[1]Mammalian Genetics Project, [4]Advanced Technical Support Department & [5]Laboratory for Transgenic Technology, Tokyo Metropolitan Institute of Medical Science, 2-1-6 Kami-kitazawa, Setagaya-ku, Tokyo, 156-8506, Japan

[2]Laboratory of Animal Resources and Development, Tokyo University of Agriculture, 196 Yasaka, Abashiri, Hokkaido 099-2493, Japan

[3]Division of Experimental Animals, Nagoya University, 65 Tsurumai-cho Showa-ku, Nagoya, Aichi 466-8550, Japan

Correspondence and requests for materials should be addressed to Y.K. (email: kikkawa-ys@igakuken.or.jp)

Supplementary Methods

Detection of the integration site of the transgene. The integration site of the transgene was detected by ligation-mediated PCR[1]. Genomic DNA was isolated from the livers of wild-type and *Prestin-hDTR* mice using the Wizard Genomic DNA Purification Kit (Promega, Madison, WI, USA). DNA was digested using *Dra*I and *Pvu*II at 37°C for 90 min, ligated to the linkers (L25: 5'-GCG GTG ACC CGG GAG ATC TGA ATT C-3' and L11: 5'-GAA TTC AGA TC-3') at 16°C overnight, and incubated at 95°C for 5 min. The sample was amplified by PCR using a KOD FX Neo instrument (TOYOBO, Osaka, Japan), the β-globin_F1 primer (CCC CTG CTG TCC ATT CCT TAT TCC ATA G) designed for the transgene construct and L25 linker primers (1/10 volume of the β-globin_F1 primer) with the following conditions: 40 cycles of 98°C for 10 sec and 68°C for 2 min. The PCR product was diluted 1:100 with distilled water and amplified by PCR using a KOD FX Neo instrument, the β-globin_F2 primer (TCC TGA CTA CTC CCA GTC ATA GCT GTC C) and L25 linker primers with the same conditions as those used for the first PCR. The second PCR product was purified using the QIAquick Gel Extraction Kit (QIAGEN, Valencia, CA, USA) and sequenced. The sequence data were aligned with the mouse genome sequence using BLAST. The integration site was confirmed by PCR using Takara Ex Taq (Takara Bio Inc., Kyoto, Japan) and the β-globin_F2, RP23-399K3_F (AGT CCC TTT TCT CAG CCT CAG CTG AC), and RP23-399K3_R (CAG AAA GGG AGT CTC AAT GAG GC) primers.

Supplementary Figures

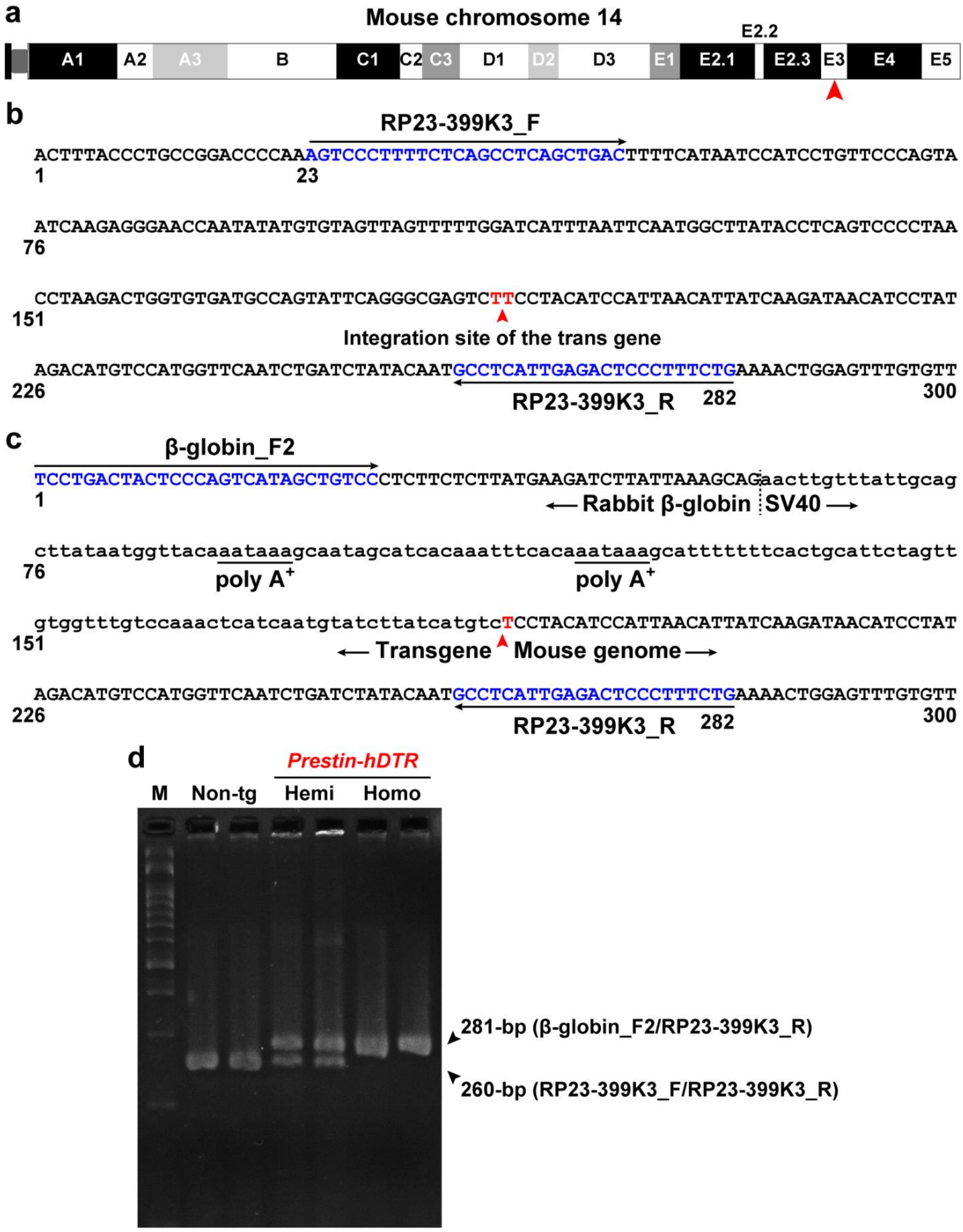

Supplementary Fig. S1. Detection of a chromosomal integration site of the transgene in *Prestin-hDTR* mice. (**a**) Diagram of mouse chromosome 14 and an integration site (red arrowhead) of the transgene. The illustration of the cytogenetic map was modified from Ensembl[2]. (**b** and **c**) Genome sequences in the vicinity of the integration site of the transgene in non-transgenic (non-tg) (**b**) and *Prestin-hDTR* (tg) (**c**) mice. Although the genome sequence in the vicinity of the integration site is not annotated in public genome databases, this sequence was included within a BAC clone (RP23-399K3) (GenBank: AC154574.2), which is mapped on the E3 region of mouse chromosome 14[3]. Nucleotide sequences of the integration site and primers used for genotyping of non-tg and tg mice are highlighted in red and blue, respectively. (**d**) Genotyping of non-tg, hemizygous tg (Hemi), and homozygous tg (Homo) mice. PCR using the RP23-399K3_F/RP23-399K3_R primer set amplified a band at 259 bp in non-tg and Hemi mice, whereas PCR using the β-globin_F2/RP23-399K3_R primer set amplified a band at 281 bp in Hemi and Homo mice. M: marker (100-bp ladder).

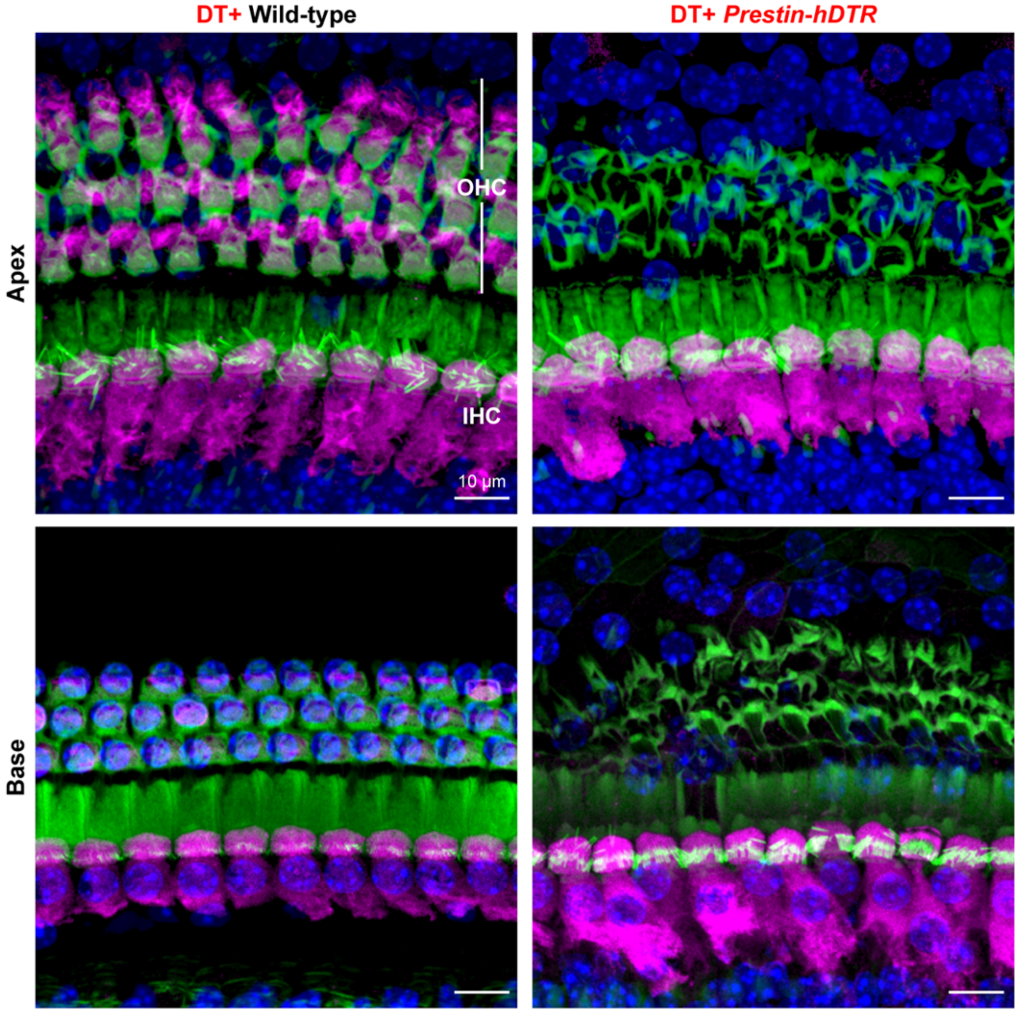

Supplementary Fig. S2. Confocal images of the hair cells from the apical and basal areas of the cochleae of DT-treated (DT+) wild-type and *Prestin-hDTR* mice. The cochleae of *Prestin-hDTR* mice were stained with anti-myosin VI (MYO6) antibody (magenta), phalloidin (green), and DAPI (blue).

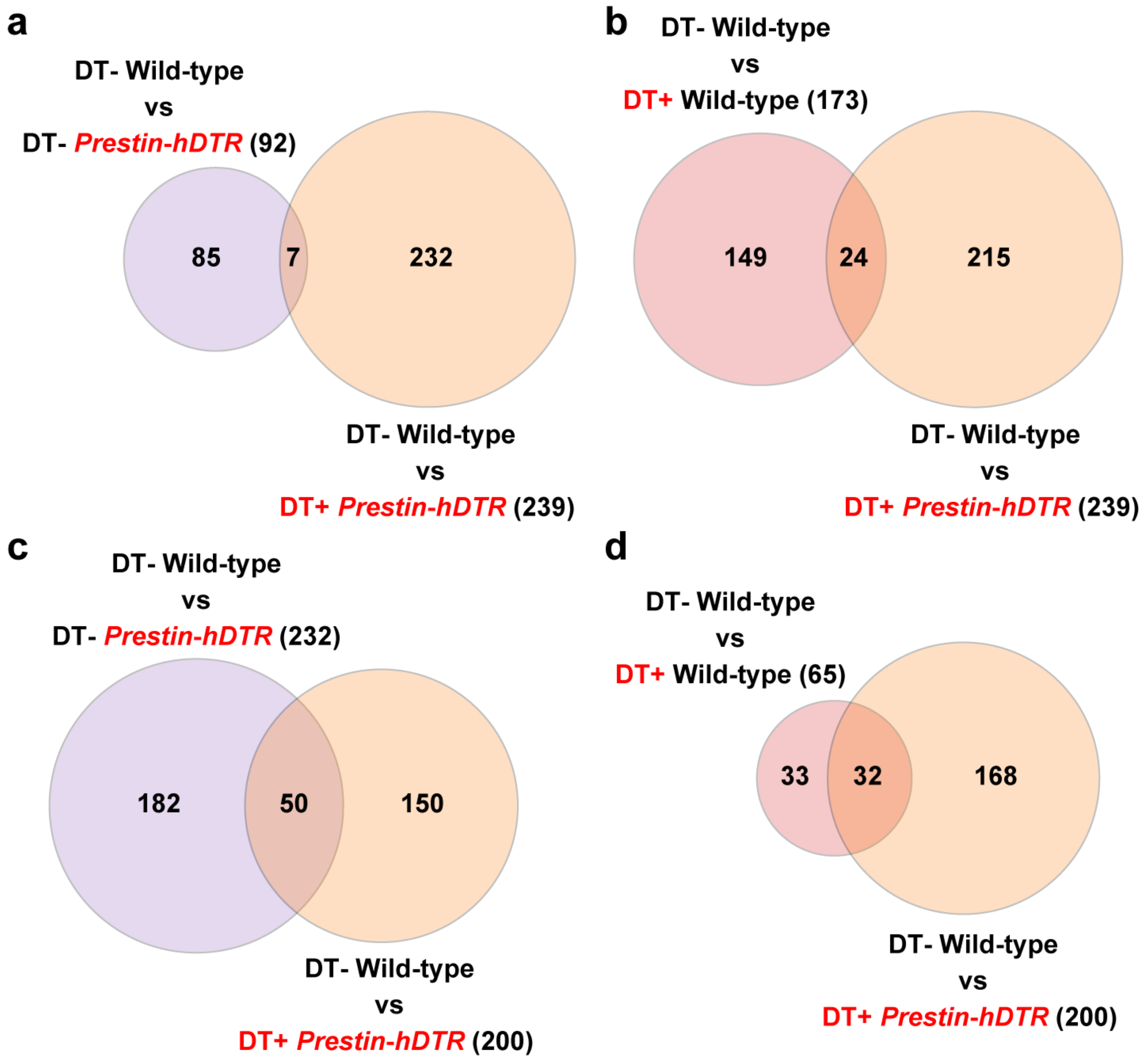

Supplementary Fig. S3. Effects of transgene (**a**, **c**) and DT administration (**b**, **d**) on gene expression in mice, as detected by microarray analysis. Venn diagrams illustrate differentially up- (**a**, **b**) and downregulated (**c**, **d**) genes (fold change $\geq \pm 2$) in cochlear RNA of untreated (DT-) wild-type vs DT- *Prestin-hDTR* and DT- wild-type vs DT-treated (DT+) *Prestin-hDTR* mice (**a**, **c**) and of DT- wild-type vs DT+ wild-type and DT- wild-type and DT+ *Prestin-hDTR* mice (**b**, **d**).

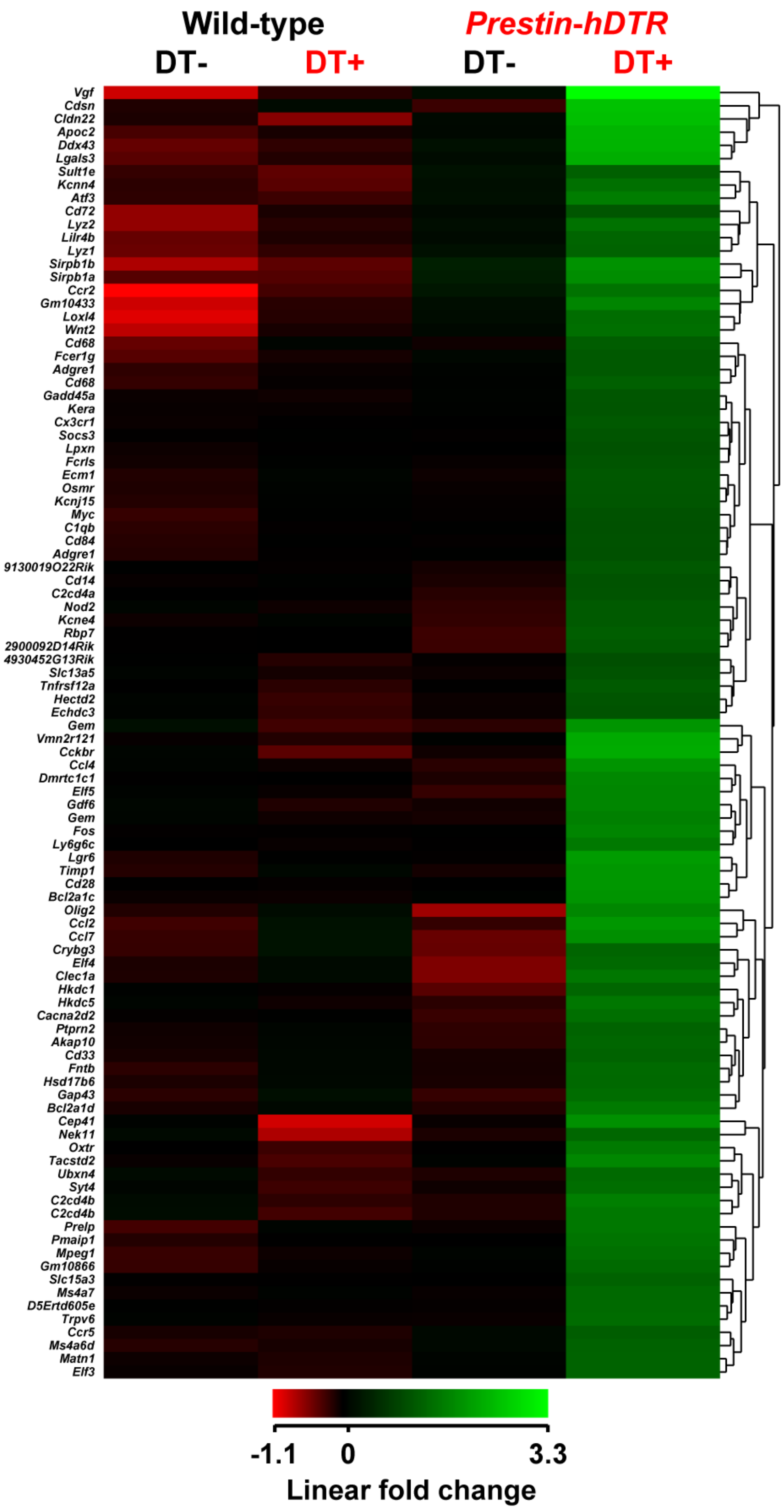

Wild-type
Prestin-hDTR
DT-
DT+
DT-
DT+
Vgf
Cdsn
Cldn22
Apoc2
Ddx43
Lgals3
Sult1e
Kcnn4
Atf3
Cd72
Lyz2
Lilr4b
Lyz1
Sirpb1b
Sirpb1a
Ccr2
Gm10433
Loxl4
Wnt2
Cd68
Fcer1g
Adgre1
Cd68
Gadd45a
Kera
Cx3cr1
Socs3
Lpxn
Fcrls
Ecm1
Osmr
Kcnj15
Myc
C1qb
Cd84
Adgre1
9130019O22Rik
Cd14
C2cd4a
Nod2
Kcne4
Rbp7
2900092D14Rik
4930452G13Rik
Slc13a5
Tnfrsf12a
Hectd2
Echdc3
Gem
Vmn2r121
Cckbr
Ccl4
Dmrtc1c1
Elf5
Gdf6
Gem
Fos
Ly6g6c
Lgr6
Timp1
Cd28
Bcl2a1c
Olig2
Ccl2
Ccl7
Crybg3
Elf4
Clec1a
Hkdc1
Hkdc5
Cacna2d2
Ptprn2
Akap10
Cd33
Fntb
Hsd17b6
Gap43
Bcl2a1d
Cep41
Nek11
Oxtr
Tacstd2
Ubxn4
Syt4
C2cd4b
C2cd4b
Prelp
Pmaip1
Mpeg1
Gm10866
Slc15a3
Ms4a7
D5Ertd605e
Trpv6
Ccr5
Ms4a6d
Matn1
Elf3
-1.1
0
3.3
Linear fold change

Supplementary Fig. S4. Heat map showing expression profiles of 98 common probes (94 genes) upregulated in *Prestin-hDTR* mice. The 98 common probes were isolated by comparison of differentially expressed genes in the cochlear RNA of untreated (DT-) wild-type vs DT-treated (DT+) *Prestin-hDTR*, DT- *Prestin-hDTR* vs DT+ *Prestin-hDTR*, and DT+ wild-type vs DT+ *Prestin-hDTR* mice (**Fig. 7b**).

Supplementary Tables

Supplementary Table S1. List of genes of upregulated genes with ≥ 2-fold change in the cochlea RNA of DT+ *Prestin-hDTR* mice.

Gene Symbol	Description [RefSeq]	Entrez Gene ID	Probe Name	Fold change
				DT+/DT-
Vgf	Mus musculus VGF nerve growth factor inducible (Vgf), mRNA [NM_001039385]	381677	A_55_P2085060	18.38
Ddx43	Mus musculus DEAD (Asp-Glu-Ala-Asp) box polypeptide 43 (Ddx43), mRNA [NM_001191044]	100048658	A_55_P2005060	6.79
Apoc2	Mus musculus apolipoprotein C-II (Apoc2), transcript variant 1, mRNA [NM_001277944]	11813	A_51_P334979	6.31
Cdsn	Mus musculus corneodesmosin (Cdsn), mRNA [NM_001008424]	386463	A_55_P2190994	6.26
Sirpb1b	Mus musculus signal-regulatory protein beta 1B (Sirpb1b), mRNA [NM_001173460]	668101	A_55_P2033041	6.23
Lgals3	Mus musculus lectin, galactose binding, soluble 3 (Lgals3), transcript variant 1, mRNA [NM_001145953]	16854	A_55_P2171116	6.22
Gm10433	Mus musculus predicted gene 10433 (Gm10433), long non-coding RNA [NR_045282]	100038378	A_55_P2062558	6.10
Ccr2	Mus musculus chemokine (C-C motif) receptor 2 (Ccr2), mRNA [NM_009915]	12772	A_51_P245989	6.06
Cldn22	Mus musculus claudin 22 (Cldn22), mRNA [NM_029383]	75677	A_55_P2112459	6.02
Loxl4	Mus musculus lysyl oxidase-like 4 (Loxl4), transcript variant 1, mRNA [NM_001164311]	67573	A_55_P2044143	5.24
Wnt2	Mus musculus wingless-type MMTV integration site family, member 2 (Wnt2), mRNA [NM_023653]	22413	A_55_P2065059	4.78
Ccl2	Mus musculus chemokine (C-C motif) ligand 2 (Ccl2), mRNA [NM_011333]	20296	A_51_P286737	4.70
Vmn2r121	Mus musculus vomeronasal 2, receptor 121 (Vmn2r121), mRNA [NM_001100616]	100038941	A_55_P2090152	4.69
Sirpb1a	Mus musculus signal-regulatory protein beta 1A (Sirpb1a), transcript variant 3, mRNA [NM_001002898]	320832	A_55_P1955308	4.60
Cckbr	Mus musculus cholecystokinin B receptor (Cckbr), mRNA [NM_007627]	12426	A_52_P596375	4.48
Lgr6	leucine-rich repeat-containing G protein-coupled receptor 6 [Source:MGI Symbol;Acc:MGI:2441805] [ENSMUST00000044828]	329252	A_52_P75777	4.44
Timp1	Mus musculus tissue inhibitor of metalloproteinase 1 (Timp1), transcript variant 1, mRNA [NM_001044384]	21857	A_55_P1985850	4.32
Lyz2	Mus musculus lysozyme 2 (Lyz2), mRNA [NM_017372]	17105	A_51_P321150	4.31
Ccl7	Mus musculus chemokine (C-C motif) ligand 7 (Ccl7), mRNA [NM_013654]	20306	A_51_P436652	4.25
Cd28	Mus musculus CD28 antigen (Cd28), mRNA [NM_007642]	12487	A_55_P1979491	3.90

Bcl2a1c	Mus musculus B cell leukemia/lymphoma 2 related protein A1c (Bcl2a1c), mRNA [NM_007535]	12046	A_52_P299771	3.88
Olig2	Mus musculus oligodendrocyte transcription factor 2 (Olig2), mRNA [NM_016967]	50913	A_52_P223626	3.72
Ccl4	Mus musculus chemokine (C-C motif) ligand 4 (Ccl4), mRNA [NM_013652]	20303	A_51_P509573	3.64
Prelp	Mus musculus proline arginine-rich end leucine-rich repeat [NM_054077]	116847	A_55_P2144736	3.59
Cep41	Mus musculus centrosomal protein 41 (Cep41), transcript variant 1, mRNA [NM_031998]	83922	A_52_P122673	3.55
Atf3	Mus musculus activating transcription factor 3 (Atf3), mRNA [NM_007498]	11910	A_52_P452689	3.52
Tacstd2	Mus musculus tumor-associated calcium signal transducer 2 (Tacstd2), mRNA [NM_020047]	56753	A_51_P257938	3.44
Dmrtc1c1	Mus musculus DMRT-like family C1c1 (Dmrtc1c1), mRNA [NM_001142691]	71083	A_55_P2180292	3.38
Gem	Mus musculus GTP binding protein (gene overexpressed in skeletal muscle) (Gem), mRNA [NM_010276]	14579	A_55_P2179027	3.36
Cd72	Mus musculus CD72 antigen (Cd72), transcript variant 1, mRNA [NM_001110320]	12517	A_55_P2180415	3.36
Lilr4b	Mus musculus leukocyte immunoglobulin-like receptor, subfamily B, member 4B (Lilr4b), transcript variant 2, mRNA [NM_001291892]	14727	A_55_P2063736	3.35
Lyz1	Mus musculus lysozyme 1 (Lyz1), mRNA [NM_013590]	17110	A_55_P2181738	3.34
Fos	Mus musculus FBJ osteosarcoma oncogene (Fos), mRNA [NM_010234]	14281	A_52_P262219	3.32
Elf5	Mus musculus E74-like factor 5 (Elf5), transcript variant 1, mRNA [NM_010125]	13711	A_51_P455866	3.24
Mpeg1	Mus musculus macrophage expressed gene 1 (Mpeg1), mRNA [NM_010821]	17476	A_51_P390538	3.22
Bcl2a1d	Mus musculus B cell leukemia/lymphoma 2 related protein A1d (Bcl2a1d), mRNA [NM_007536]	12047	A_55_P1978424	3.21
Pmaip1	Mus musculus phorbol-12-myristate-13-acetate-induced protein 1 (Pmaip1), mRNA [NM_021451]	58801	A_51_P477121	3.16
Clec1a	Mus musculus C-type lectin domain family 1, member a (Clec1a), transcript variant 1, mRNA [NM_175526]	243653	A_52_P624434	3.16
Gdf6	Mus musculus growth differentiation factor 6 (Gdf6), mRNA [NM_013526]	242316	A_52_P28960	3.16
Cd68	CD68 antigen [Source:MGI Symbol;Acc:MGI: 88342] [ENSMUST00000108654]	12514	A_55_P2067583	3.11
Kcnn4	Mus musculus potassium intermediate/small conductance calcium-activated channel, subfamily N, member 4 (Kcnn4), transcript variant 1, mRNA [NM_008433]	16534	A_51_P389636	3.11
Slc15a3	Mus musculus solute carrier family 15, member 3 (Slc15a3), mRNA [NM_023044]	65221	A_52_P467389	3.04
Gap43	Mus musculus growth associated protein 43 (Gap43), mRNA [NM_008083]	14432	A_55_P2096867	3.02

Gem	Mus musculus GTP binding protein (gene overexpressed in skeletal muscle) (Gem), mRNA [NM_010276]	14579	A_55_P2111790	3.01
Oxtr	Mus musculus oxytocin receptor (Oxtr), mRNA [NM_001081147]	18430	A_55_P2013203	2.99
Ly6g6c	Mus musculus lymphocyte antigen 6 complex, locus G6C (Ly6g6c), mRNA [NM_023463]	68468	A_55_P2141395	2.96
Fntb	Mus musculus farnesyltransferase, CAAX box, beta (Fntb), mRNA [NM_145927]	110606	A_55_P2076852	2.96
Crybg3	PREDICTED: Mus musculus beta-gamma crystallin domain containing 3 (Crybg3), transcript variant X1, mRNA [XM_006522095]	224273	A_55_P2052709	2.91
Fcer1g	Mus musculus Fc receptor, IgE, high affinity I, gamma polypeptide (Fcer1g), mRNA [NM_010185]	14127	A_51_P405476	2.89
C2cd4b	Mus musculus C2 calcium-dependent domain containing 4B (C2cd4b), mRNA [NM_001081314]	75697	A_51_P415395	2.86
Hdhd5	Mus musculus cat eye syndrome chromosome region, candidate 5 (Cecr5), mRNA [NM_144815]	214932	A_52_P565279	2.80
Hsd17b6	Mus musculus hydroxysteroid (17-beta) dehydrogenase 6 (Hsd17b6), mRNA [NM_013786]	27400	A_52_P590154	2.79
Elf4	Mus musculus E74-like factor 4 (ets domain transcription factor) (Elf4), mRNA [NM_019680]	56501	A_51_P448856	2.78
Cd68	Mus musculus CD68 antigen (Cd68), transcript variant 1, mRNA [NM_001291058]	12514	A_55_P1961943	2.77
Sult1e1	Mus musculus sulfotransferase family 1E, member 1 (Sult1e1), mRNA [NM_023135]	20860	A_51_P493649	2.77
Cacna2d2	Mus musculus calcium channel, voltage-dependent, alpha 2/delta subunit 2 (Cacna2d2), transcript variant 1, mRNA [NM_001174047]	56808	A_55_P2122879	2.75
Ms4a6d	Mus musculus membrane-spanning 4-domains, subfamily A, member 6D (Ms4a6d), mRNA [NM_026835]	68774	A_51_P149714	2.71
C2cd4b	Mus musculus C2 calcium-dependent domain containing 4B (C2cd4b), mRNA [NM_001081314]	75697	A_55_P2054261	2.68
Ms4a7	Mus musculus membrane-spanning 4-domains, subfamily A, member 7 (Ms4a7), transcript variant 3, mRNA [NM_001276398]	109225	A_55_P1958165	2.66
Akap10	Mus musculus A kinase (PRKA) anchor protein 10 (Akap10), mRNA [NM_019921]	56697	A_52_P75866	2.63
Cd33	Mus musculus CD33 antigen (Cd33), transcript variant 1, mRNA [NM_001111058]	12489	A_66_P109986	2.60
Trpv6	Mus musculus transient receptor potential cation channel, subfamily V, member 6 (Trpv6), mRNA [NM_022413]	64177	A_55_P2111444	2.59
Syt4	Mus musculus synaptotagmin IV (Syt4), mRNA [NM_009308]	20983	A_52_P628915	2.58
D5Ertd605e	Mus musculus DNA segment, Chr 5, ERATO Doi 605, expressed (D5Ertd605e), long non-coding RNA [NR_033625]	100039805	A_55_P2035326	2.56

Ptprn2	Mus musculus protein tyrosine phosphatase, receptor type, N polypeptide 2 (Ptprn2), mRNA [NM_011215]	19276	A_55_P1977369	2.55
Adgre1	Mus musculus adhesion G protein-coupled receptor E1 (Adgre1), mRNA [NM_010130]	13733	A_55_P2018934	2.55
Matn1	Mus musculus matrilin 1, cartilage matrix protein (Matn1), mRNA [NM_010769]	17180	A_55_P2171221	2.53
Elf3	Mus musculus E74-like factor 3 (Elf3), transcript variant 2, mRNA [NM_007921]	13710	A_51_P480241	2.49
Hkdc1	Mus musculus hexokinase domain containing 1 (Hkdc1), mRNA [NM_145419]	216019	A_51_P322972	2.47
Ccr5	Mus musculus chemokine (C-C motif) receptor 5 (Ccr5), mRNA [NM_009917]	12774	A_52_P578732	2.46
Myc	Mus musculus myelocytomatosis oncogene (Myc), transcript variant 1, mRNA [NM_010849]	17869	A_52_P108346	2.45
Ecm1	Mus musculus extracellular matrix protein 1 (Ecm1), transcript variant 1, mRNA [NM_007899]	13601	A_66_P104815	2.45
Gm10866	PREDICTED: Mus musculus predicted gene 10866 (Gm10866), misc_RNA [XR_141279]	100038696	A_55_P2157537	2.42
Kcnj15	Mus musculus potassium inwardly-rectifying channel, subfamily J, member 15 (Kcnj15), transcript variant 2, mRNA [NM_019664]	16516	A_55_P2164090	2.40
Osmr	Mus musculus oncostatin M receptor (Osmr), transcript variant 1, mRNA [NM_011019]	18414	A_51_P319460	2.39
Ubxn4	Mus musculus UBX domain protein 4 (Ubxn4), mRNA [NM_026390]	67812	A_52_P401475	2.36
C1qb	Mus musculus complement component 1, q subcomponent, beta polypeptide (C1qb), mRNA [NM_009777]	12260	A_51_P351860	2.35
Kcne4	Mus musculus potassium voltage-gated channel, Isk-related subfamily, gene 4 (Kcne4), mRNA [NM_021342]	57814	A_51_P508838	2.34
Rbp7	Mus musculus retinol binding protein 7, cellular (Rbp7), mRNA [NM_022020]	63954	A_52_P343627	2.34
Cd84	Mus musculus CD84 antigen (Cd84), transcript variant 1, mRNA [NM_013489]	12523	A_55_P2054445	2.33
Nek11	Mus musculus NIMA (never in mitosis gene a)-related expressed kinase 11 (Nek11), mRNA [NM_172461]	208583	A_51_P262325	2.31
Adgre1	Mus musculus adhesion G protein-coupled receptor E1 (Adgre1), mRNA [NM_010130]	13733	A_55_P2144386	2.29
Cx3cr1	Mus musculus chemokine (C-X3-C motif) receptor 1 (Cx3cr1), mRNA [NM_009987]	13051	A_52_P99810	2.28
Fcrls	Mus musculus Fc receptor-like S, scavenger receptor (Fcrls), mRNA [NM_030707]	80891	A_52_P231075	2.25
2900092D14Rik	Mus musculus RIKEN cDNA 2900092D14 gene (2900092D14Rik), long non-coding RNA [NR_027891]	73100	A_55_P2319478	2.25
Tnfrsf12a	Mus musculus tumor necrosis factor receptor superfamily, member 12a (Tnfrsf12a), transcript variant 1, mRNA [NM_013749]	27279	A_51_P131408	2.23

Gadd45a	Mus musculus growth arrest and DNA-damage-inducible 45 alpha (Gadd45a), mRNA [NM_007836]	13197	A_51_P296608	2.23
Kera	Mus musculus keratocan (Kera), mRNA [NM_008438]	16545	A_51_P123655	2.21
9130019O22Rik	Mus musculus RIKEN cDNA 9130019O22 gene (9130019O22Rik), mRNA [NM_030226]	78921	A_55_P2044827	2.17
Lpxn	Mus musculus leupaxin (Lpxn), mRNA [NM_134152]	107321	A_52_P577748	2.17
Socs3	Mus musculus suppressor of cytokine signaling 3 (Socs3), mRNA [NM_007707]	12702	A_51_P474459	2.16
Cd14	Mus musculus CD14 antigen (Cd14), mRNA [NM_009841]	12475	A_51_P172853	2.16
Nod2	Mus musculus nucleotide-binding oligomerization domain containing 2 (Nod2), mRNA [NM_145857]	257632	A_55_P2359797	2.16
C2cd4a	Mus musculus C2 calcium-dependent domain containing 4A (C2cd4a), mRNA [NM_001163143]	244911	A_55_P2147897	2.12
Hectd2	Mus musculus HECT domain containing 2 (Hectd2), transcript variant 2, mRNA [NM_172637]	226098	A_52_P502141	2.07
Slc13a5	Mus musculus solute carrier family 13 (sodium-dependent citrate transporter), member 5 (Slc13a5), mRNA [NM_001004148]	237831	A_55_P1963508	2.04
Echdc3	enoyl Coenzyme A hydratase domain containing 3 [Source:MGI Symbol;Acc:MGI:1915106] [ENSMUST00000114941]	67856	A_55_P2131190	2.04
4930452G13Rik	Mus musculus RIKEN cDNA 4930452G13 gene (4930452G13Rik), long non-coding RNA [NR_045060]	73989	A_55_P2204168	2.01

Supplementary Table S2. Gene ontological categories of upregulated genes with statistical significance ($P < 0.05$) in the cochlea RNA of DT+ *Prestin-hDTR* mice.

**GO accession no.**	**GO term**	***P*-value**	**Gene Symbol**
GO:0032760	Positive regulation of tumor necrosis factor production	1.62E-04	*Ccl2*, *Ccl4*, *Ccr2*, *Ccr5*, *Cd14*, *Fcer1g*, *Nod2*
GO:1903557	Positive regulation of tumor necrosis factor superfamily cytokine production	1.62E-04	*Ccl2*, *Ccl4*, *Ccr2*, *Ccr5*, *Cd14*, *Fcer1g*, *Nod2*
GO:0097529	Myeloid leukocyte migration	5.63E-04	*Ccl2*, *Ccl4*, *Ccl7*, Ccr2, *Cx3cr1*, *Fcer1g*, *Lgals3*
GO:0006950	Response to stress	9.70E-04	*Atf3*, *Bcl2a1c*, *Bcl2a1d*, *C1qb*, *Cckbr*, *Ccl2*, *Ccl4*, *Ccl7*, *Ccr2*, *Ccr5*, *Cd14*, *Cd84*, *Ecm1*, *Elf3*, *Elf4*, *Fcer1g*, *Fntb*, *Fos*, *Gadd45a*, *Lgals3*, *Lgr6*, *Lyz1*, *Lyz2*, *Myc*, *Nek11*, *Nod2*, *Oxtr*, *Pmaip1*, *Ubxn4*, *Vgf*
GO:0070098	Chemokine-mediated signaling pathway	9.70E-04	*Ccl2*, *Ccl4*, *Ccl7*, *Ccr2*, *Ccr5*, *Cx3cr1*
GO:0048522|GO:0051242	Positive regulation of cellular process	1.49E-03	*Apoc2*, *Atf3*, *Bcl2a1c*, *Bcl2a1d*, *Cckbr*, *Ccl2*, *Ccl4*, *Ccl7*, *Ccr2*, *Ccr5*, *Cd14*, *Cd28*, *Cd84*, *Cx3cr1*, *Ecm1*, *Elf3*, *Elf4*, *Elf5*, *Fcer1g*, *Fntb*, *Fos*, *Gadd45a*, *Gdf6*, *Kcnn4*, *Lgals3*, *Lgr6*, *Myc*, *Nod2*, *Olig2*, *Osmr*, *Oxtr*, *Pmaip1*, *Sirpb1a*, *Socs3*, *Sult1e1*, *Syt4*, *Tacstd2*, *Timp1*, *Tnfrsf12a, Wnt2*
GO:0030595	Leukocyte chemotaxis	1.49E-03	*Ccl2*, *Ccl4*, *Ccl7*, *Ccr2*, *Cx3cr1*, *Fcer1g*, *Lgals3*
GO:1903555	Regulation of tumor necrosis factor superfamily cytokine production	1.52E-03	*Ccl2*, *Ccl4*, *Ccr2*, *Ccr5*, *Cd14*, *Fcer1g*, *Nod2*
GO:0006952|0002217|0042829	Defense response	1.52E-03	*C1qb*, *Cckbr*, *Ccl2*, *Ccl4*, *Ccl7*, *Ccr2*, *Ccr5*, *Cd14*, *Cd84*, *Ecm1*, *Elf3*, *Elf4*, *Fcer1g*, *Lgals3*, *Lyz1,* *Lyz2*, *Nod2*
GO:0032680	Regulation of tumor necrosis factor production	1.52E-03	*Cd14, Ccr2, Ccl2, Fcer1g, Ccl4, Ccr5, Nod2*
GO:0048518|0043119	Positive regulation of biological process	1.63E-03	*Apoc2*, *Atf3*, *Bcl2a1c*, *Bcl2a1d*, *Cacna2d2*, *C1qb*, *Cckbr*, *Ccl2*, *Ccl4*, *Ccl7*, *Ccr2*, *Ccr5*, *Cd14*, *Cd28*, *Cd84*, *Cx3cr1*, *Elf3*, *Elf4*, *Elf5*, *Ecm1*, *Fcer1g*, *Fntb*, *Fos*, *Gadd45a*, *Gdf6*, *Kcnn4*, *Lgals3*, *Lgr6*, *Myc*, *Nod2*, *Olig2*, *Osmr*, *Oxtr*, *Pmaip1*, *Sirpb1a*, *Socs3*, *Sult1e1*, *Syt4*, *Tacstd2*, *Timp1*, *Tnfrsf12a*, *Wnt2*
GO:0006955	Immune response	2.75E-03	*Cd14*, *Ccr2*, *Ccl2*, *C1qb*, *Fcer1g*, *Ccl7*, *Elf4*, *Ccl4*, *Ccr5*, *Cx3cr1*, *Cd28*, *Adgre1*, *Cd84*, *Adgre1*, *Lgals3*, *Nod2*

GO:0034097	Response to cytokine	6.07E-03	*Ccl2*, *Ccl4*, *Ccl7*, *Ccr2*, *Ccr5*, *Cd14*, *Cx3cr1*, *Fntb, Myc*, *Osmr*, *Socs3*, *Timp1*
GO:0002548	Monocyte chemotaxis	6.36E-03	*Ccl4*, *Ccl7*, *Ccr2*, *Lgals3*
GO:1990266	Neutrophil migration	6.36E-03	*Ccl2*, *Ccl4*, *Ccl7*, *Fcer1g*, *Lgals3*
GO:0030593	Neutrophil chemotaxis	6.36E-03	*Ccl2*, *Ccl4*, *Ccl7*, *Fcer1g*, *Lgals3*
GO:0009897	External side of plasma membrane	6.36E-03	*Adgre1*, *Ccr2*, *Ccr5*, *Cd14*, *Cd33*, *Cd28*, *Fcer1g*, *Lgals3*, *Osmr*
GO:0050776	Regulation of immune response	6.40E-03	*Bcl2a1d*, *C1qb*, *Ccr2*, *Cd14*, *Cd28*, *Cd84*, *Ecm1*, *Fcer1g*, *Kcnn4*, *Lgals3*, *Lpxn*, *Nod2*
GO:0050900	Leukocyte migration	6.49E-03	*Ccl2*, *Ccl7*, *Ccl4*, *Ccr2*, *Cx3cr1*, *Fcer1g*, *Lgals3*
GO:0002684	Positive regulation of immune system process	6.49E-03	*Bcl2a1d*, *C1qb*, *Ccl2*, *Ccl4*, *Ccr2*, *Cd14*, *Cd28*, *Cd84*, *Fcer1g*, *Fos*, *Kcnn4*, *Lgals3*, *Nod2*
GO:0060326	Cell chemotaxis	6.66E-03	*Ccl2*, *Ccl4*, *Ccl7*, *Ccr2*, *Cx3cr1*, *Fcer1g*, *Lgals3*
GO:0071674	Mononuclear cell migration	7.50E-03	*Ccl4*, *Ccl7*, *Ccr2*, *Lgals3*
GO:0071621	Granulocyte chemotaxis	7.50E-03	*Ccl2*, *Ccl4*, *Ccl7*, *Fcer1g*, *Lgals3*
GO:0006954	Inflammatory response	7.50E-03	*Ccl2*, *Ccl4*, *Ccl7*, *Ccr2*, *Ccr5*, *Cd14*, *Ecm1*, *Elf3*, *Fcer1g*, *Nod2*
GO:0008284	Positive regulation of cell proliferation	7.60E-03	*Atf3*, *Ccl2*, *Cckbr*, *Ccr2*, *Cd28*, *Cx3cr1*, *Ecm1*, *Fntb*, *Lgals3*, *Myc*, *Nod2*, *Osmr*, *Timp1*, *Wnt2*
GO:0097530	Granulocyte migration	7.60E-03	*Ccl2*, *Ccl4*, *Ccl7*, *Fcer1g*, *Lgals3*
GO:0051240	Positive regulation of multicellular organismal process	9.11E-03	*Cacna2d2*, *Ccl2*, *Ccl4*, *Ccr2*, *Ccr5*, *Cd14*, *Cd28*, *Cd84*, *Cx3cr1*, *Ecm1*, *Fcer1g*, *Fos*, *Gdf6*, *Lgals3*, *Nod2*, *Olig2*, *Oxtr*, *Tnfrsf12a*, *Wnt2*
GO:0007166	Cell surface receptor signaling pathway	9.11E-03	*Adgre1*, *Bcl2a1c*, *Bcl2a1d*, *Ccl2*, *Ccl4*, *Ccl7*, *Ccr2*, *Ccr5*, *Cd14*, *Cd28*, *Cx3cr1*, *Fcer1g*, *Fos*, *Gdf6*, *Lgr6*, *Myc*, *Osmr*, *Socs3*, *Tnfrsf12a*, *Wnt2*
GO:0048247	Lymphocyte chemotaxis	1.19E-02	*Ccl2*, *Ccl4*, *Ccl7*, *Ccr2*
GO:0045087|0002226	Innate immune response	1.19E-02	*C1qb*, *Ccl2*, *Ccl4*, *Ccl7*, *Cd14*, *Cd84*, *Elf4*, *Fcer1g*, *Lgals3*, *Nod2*
GO:0090264	Regulation of immune complex clearance by monocytes and macrophages	1.19E-02	*Ccl2*, *Ccr2*
GO:0090265	Positive regulation of immune complex clearance by monocytes and macrophages	1.19E-02	*Ccl2*, *Ccr2*
GO:0001819	Positive regulation of cytokine production	1.19E-02	*Ccl2*, *Ccl4*, *Ccr2*, *Ccr5*, *Cd14*, *Cd28*, *Cd84*, *Fcer1g*, *Nod2*
GO:0032701	Negative regulation of interleukin-18 production	1.19E-02	*Cd84*, *Nod2*

GO:0048584	Positive regulation of response to stimulus	1.19E-02	*Bcl2a1d*, *C1qb*, *Ccl2*, *Ccl4*, *Ccl7*, *Ccr2*, *Ccr5*, *Cd14*, *Cd28*, *Cd84*, *Ecm1*, *Fcer1g*, *Gadd45a*, *Gdf6*, *Kcnn4*, *Lgr6*, *Nod2*, *Pmaip1*, *Tnfrsf12a*, *Wnt2*
GO:0032879	Regulation of localization	1.42E-02	*Apoc2*, *Cacna2d2*, *Cckbr*, *Ccl2*, *Ccl4*, *Ccr2*, *Ccr5*, *Cd14*, *Cd84*, *Cx3cr1*, *Ecm1*, *Fcer1g*, *Kcne4*, *Kcnj15*, *Kcnn4*, *Lgals3*, *Lgr6*, *Myc*, *Oxtr*, *Pmaip1*, *Sirpb1a*, *Syt4*, *Tacstd2*
GO:0030100	Regulation of endocytosis	1.42E-02	*Apoc2*, *Ccl2*, *Cd14*, *Fcer1g*, *Lgals3*, *Sirpb1a*, *Syt4*
GO:1905517	Macrophage migration	1.48E-02	*Ccl2*, *Cx3cr1*, *Lgals3*
GO:0048246	Macrophage chemotaxis	1.48E-02	*Ccl2*, *Cx3cr1*, *Lgals3*
GO:0032653	Regulation of interleukin-10 production	1.60E-02	*Cd28*, *Cd84*, *Fcer1g*, *Nod2*
GO:0045765	Regulation of angiogenesis	1.60E-02	*Ccl2*, *Ccr2*, *Ccr5*, *Cx3cr1*, *Ecm1*, *Lgals3*, *Tnfrsf12a*
GO:0098552	Side of membrane	1.64E-02	*Adgre1*, *Ccr2*, *Ccr5*, *Cd14*, *Cd28*, *Cd33, Fcer1g*, *Gem*, *Lgals3*, *Osmr*
GO:0002682	Regulation of immune system process	1.64E-02	*Bcl2a1d*, *C1qb*, *Ccl2*, *Ccl4*, *Ccr2*, *Cd14*, *Cd28*, *Cd84*, *Ecm1*, *Fcer1g*, *Fos*, *Kcnn4*, *Lgals3*, *Lpxn*, *Nod2*
GO:0010033	Response to organic substance	1.79E-02	*Atf3*, *Ccl2*, *Ccl4*, *Ccl7*, *Ccr2*, *Ccr5*, *Cd14*, *Cd68*, *Cx3cr1*, *Fcer1g*, *Fntb*, *Fos*, *Gdf6*, *Myc*, *Nod2*, *Osmr*, *Oxtr*, *Ptprn2*, *Socs3*, *Timp1*, *Ubxn4*, *Vgf*
GO:1901342	Regulation of vasculature development	2.00E-02	*Ccl2*, *Ccr2*, *Ccr5*, *Cx3cr1*, *Ecm1*, *Lgals3*, *Tnfrsf12a*
GO:0072676	Lymphocyte migration	2.06E-02	*Ccl2*, *Ccl4*, *Ccl7*, *Ccr2*
GO:0019221	Cytokine-mediated signaling pathway	2.08E-02	*Ccl2*, *Ccl4*, *Ccl7*, *Ccr2*, *Ccr5*, *Cx3cr1*, *Osmr*, *Socs3*
GO:0005886|GO:0005904	Plasma membrane	2.18E-02	*Adgre1*, *Akap10*, *C2cd4a*, *C2cd4b*, *Cacna2d2*, *Cckbr*, *Ccr2*, *Ccr5*, *Cd14*, *Cd28*, *Cd33*, *Cd68*, *Cd72*, *Cd84*, *Cldn22*, *Cx3cr1*, *Fcer1g*, *Gap43*, *Gem*, *Kcne4*, *Kcnj15*, *Kcnn4*, *Lgals3*, *Lgr6*, *Lpxn*, *Ly6g6c*, *Nod2*, *Osmr*, *Oxtr*, *Sirpb1a*, *Slc13a5*, *Syt4*, *Tacstd2*, *Tnfrsf12a*, *Trpv6*, *Wnt2*
GO:0051094	Positive regulation of developmental process	2.25E-02	*Cacna2d2*, *Ccr2*, *Ccr5*, *Cd28*, *Cx3cr1*, *Ecm1*, *Fos*, *Gdf6*, *Lgals3*, *Olig2*, *Oxtr*, *Socs3*, *Sult1e1*, *Tacstd2*, *Tnfrsf12a*, *Wnt2*
GO:0051049	Regulation of transport	2.27E-02	*Apoc2*, *Cacna2d2*, *Cckbr*, *Ccl2*, *Ccl4*, *Ccr2*, *Ccr5*, *Cd14*, *Cd84*, *Fcer1g*, *Kcne4*, *Kcnj15*, *Kcnn4*, *Lgals3*, *Oxtr*, *Pmaip1*, *Sirpb1a*, *Syt4*

GO:0032103	Positive regulation of response to external stimulus	2.27E-02	*Ccl2*, *Ccl4*, *Ccr2*, *Ccr5*, *Cd28*, *Fcer1g*, *Nod2*
GO:0060627	Regulation of vesicle-mediated transport	2.65E-02	*Apoc2*, *Ccl2*, *Ccr2*, *Cd14*, *Cd84*, *Fcer1g*, *Lgals3*, *Sirpb1a*, *Syt4*
GO:0006935	Chemotaxis	2.65E-02	*Ccl2*, *Ccl4*, *Ccl7*, *Ccr2*, *Ccr5*, *Cx3cr1*, *Fcer1g*, *Gap43*, *Lgals3*
GO:0042330	Taxis	2.69E-02	*Ccl2*, *Ccl4*, *Ccl7*, *Ccr2*, *Ccr5*, *Cx3cr1*, *Fcer1g*, *Gap43*, *Lgals3*
GO:0071677	Positive regulation of mononuclear cell migration	2.75E-02	*Ccl2*, *Ccr2*, *Lgals3*
GO:0071944	Cell periphery	3.11E-02	*Adgre1*, *Akap10*, *C2cd4a*, *C2cd4b*, *Cacna2d2*, *Cckbr*, *Ccr2*, *Ccr5*, *Cd14*, *Cd28*, *Cd33*, *Cd68*, *Cd72*, *Cd84*, *Cldn22*, *Cx3cr1*, *Fcer1g*, *Gap43*, *Gem*, *Kcne4*, *Kcnj15*, *Kcnn4*, *Lgals3*, *Lgr6*, *Lpxn*, *Ly6g6c*, *Nod2*, *Osmr*, *Oxtr*, *Sirpb1a*, *Slc13a5*, *Syt4*, *Tacstd2*, *Tnfrsf12a*, *Trpv6*, *Wnt2*
GO:1903306	Negative regulation of regulated secretory pathway	3.11E-02	*Ccr2*, *Cd84*, *Syt4*
GO:0002699	Positive regulation of immune effector process	3.26E-02	*Ccl2*, *Ccr2*, *Cd28*, *Cd84*, *Fcer1g*, *Nod2*
GO:0050727	Regulation of inflammatory response	3.26E-02	*Ccr2*, *Ccr5*, *Cd28*, *Cx3cr1*, *Fcer1g*, *Socs3*, *Nod2,*
GO:0048583	Regulation of response to stimulus	3.47E-02	*Atf3*, *Bcl2a1d*, *C1qb*, *Ccl2*, *Ccl4*, *Ccl7*, *Ccr2*, *Ccr5*, *Cd14*, *Cd28*, *Cd84*, *Cx3cr1*, *Ecm1*, *Fcer1g*, *Gadd45a*, *Gdf6*, *Kcnn4*, *Lgals3*, *Lgr6*, *Lpxn*, *Nod2*, *Pmaip1*, *Socs3*, *Syt4*, *Timp1*, *Tnfrsf12a*, *Vgf*, *Wnt2*
GO:0051050	Positive regulation of transport	3.53E-02	*Cckbr*, *Ccl2*, *Ccl4*, *Ccr5*, *Cd14*, *Fcer1g*, *Kcnn4*, *Lgals3*, *Oxtr*, *Pmaip1*, *Sirpb1a*, *Syt4*
GO:0045920	Negative regulation of exocytosis	3.79E-02	*Ccr2*, *Cd84*, *Syt4*
GO:0031727	CCR2 chemokine receptor binding	3.79E-02	*Ccl2*, *Ccl7*
GO:0080134	Regulation of response to stress	3.81E-02	*Ccl2*, *Ccr2*, *Ccr5*, *Cd14*, *Cd28*, *Cx3cr1*, *Fcer1g*, *Gadd45a*, *Gdf6*, *Nod2*, *Pmaip1*, *Socs3*, *Tnfrsf12a*
GO:1903532	Positive regulation of secretion by cell	4.09E-02	*Cckbr*, *Ccr5*, *Cd14*, *Fcer1g*, *Kcnn4*, *Lgals3*, *Oxtr*, *Syt4*
GO:0001836	Release of cytochrome c from mitochondria	4.14E-02	*Bcl2a1c*, *Bcl2a1d*, *Pmaip1*
GO:0046903	secretion	4.37E-02	*Cckbr*, *Fcer1g*, *Kcnn4*, *Nod2*, *Oxtr*, *Ptprn2*, *Syt4*, *Trpv6*, *Vgf*
GO:0001637	G-protein coupled chemoattractant receptor activity	4.37E-02	*Ccr2*, *Ccr5*, *Cx3cr1*
GO:0019956	Chemokine binding	4.37E-02	*Ccr2*, *Ccr5*, *Cx3cr1*
GO:0051239	Regulation of multicellular organismal process	4.37E-02	*Apoc2*, *Cacna2d2*, *Cckbr*, *Ccl2*, *Ccl4*, *Ccr2*, *Ccr5*, *Cd14*, *Cd28*, *Cd84*, *Cx3cr1*, *Ecm1*, *Fcer1g*, *Fos*, *Gdf6*, *Lgals3*, *Matn1*, *Nod2*, *Olig2*, *Oxtr*, *Syt4*, *Tacstd2*, *Tnfrsf12a*, *Wnt2*

GO:0004950	Chemokine receptor activity	4.37E-02	*Ccr2*, *Ccr5*, *Cx3cr1*
GO:0071345	Cellular response to cytokine stimulus	4.37E-02	*Ccl2*, *Ccl4*, *Ccl7*, *Ccr2*, *Ccr5*, *Cx3cr1*, *Myc*, *Osmr*, *Socs3*
GO:0042127	Regulation of cell proliferation	4.37E-02	*Atf3*, *Cckbr*, *Ccl2*, *Ccr2*, *Ccr5*, *Cd28*, *Cx3cr1*, *Ecm1*, *Fntb*, *Lgals3*, *Myc*, *Nod2*, *Osmr*, *Pmaip1*, *Tacstd2*, *Timp1*, *Wnt2*
GO:0002376	Immune system process	4.37E-02	*Adgre1*, *Bcl2a1d*, *C1qb*, *Ccl2*, *Ccl4*, *Ccl7*, *Ccr2*, *Ccr5*, *Cd14*, *Cd28*, *Cd84*, *Cx3cr1*, *Elf4*, *Fcer1g*, *Kcnn4*, *Lgals3*, *Nod2*, *Pmaip1*
GO:0042803	Protein homodimerization activity	4.49E-02	*Apoc2*, *Atf3*, *Bcl2a1c*, *C1qb*, *Cd28*, *Cdsn*, *Fcer1g*, *Gadd45a*, *Gdf6*, *Olig2*, *Syt4*
GO:0002828	Regulation of type 2 immune response	4.64E-02	*Ccr2*, *Ecm1*, *Nod2*
GO:0002253	Activation of immune response	4.71E-02	*Bcl2a1d*, *C1qb*, *Cd14*, *Cd28*, *Fcer1g*, *Nod2*

Supplementary Table S3. List of genes of downregulated genes with ≤ - 2-fold change in the cochlea RNA of DT+ *Prestin-hDTR* mice. *The GeneID was changed to 108168154.

Gene Symbol	Description [RefSeq]	Entrez Gene ID	Probe Name	Fold change
				DT+/DT-
Strc	Mus musculus stereocilin (Strc), mRNA [NM_080459]	140476	A_55_P2004001	-8.52
Chrna10	Mus musculus cholinergic receptor, nicotinic, alpha polypeptide 10 (Chrna10), mRNA [NM_001081424]	504186	A_55_P1991244	-7.60
Gm46479	PREDICTED: Mus musculus uncharacterized LOC108168195 (LOC108168195), ncRNA [XR_001781413]	108168195	A_55_P1958544	-7.25
Chrna9	Mus musculus cholinergic receptor, nicotinic, alpha polypeptide 9 (Chrna9), mRNA [NM_001081104]	231252	A_51_P381069	-6.79
Gm46479	PREDICTED: Mus musculus uncharacterized LOC108168195 (LOC108168195), ncRNA [XR_001781413]	108168195	A_55_P2051166	-5.92
Ocm	Mus musculus oncomodulin (Ocm), mRNA [NM_033039]	18261	A_55_P2094916	-5.74
Gm3161	predicted gene 3161	100041143*	A_55_P2019838	-5.51
Myo15	Mus musculus myosin XV (Myo15), transcript variant 1, mRNA [NM_010862]	17910	A_55_P1969977	-5.48
Ocm	Mus musculus oncomodulin (Ocm), mRNA [NM_033039]	18261	A_55_P2073825	-5.15
Gfi1	Mus musculus growth factor independent 1 (Gfi1), transcript variant 1, mRNA [NM_010278]	14581	A_55_P1977008	-4.20
Gm3161	predicted gene 3161	100041143*	A_55_P1957867	-4.17
Ppp1r17	Mus musculus protein phosphatase 1, regulatory subunit 17 (Ppp1r17), mRNA [NM_011153]	19051	A_52_P355124	-3.90
Lhx3	Mus musculus LIM homeobox protein 3 (Lhx3), transcript variant 1, mRNA [NM_001039653]	16871	A_55_P2031272	-3.88
Barhl1	Mus musculus BarH-like 1 (Drosophila) (Barhl1), transcript variant 1, mRNA [NM_019446]	54422	A_52_P65084	-3.86
Mkrn2os	Mus musculus makorin, ring finger protein 2, opposite strand (Mkrn2os), mRNA [NM_001101431]	70291	A_52_P512427	-3.24
Vwa3a	Mus musculus von Willebrand factor A domain containing 3A (Vwa3a), mRNA [NM_177697]	233813	A_66_P139647	-3.23
Pjvk	Mus musculus pejvakin (Pjvk), mRNA, mRNA [NM_001080711]	381375	A_55_P2181381	-3.04
Pou4f3	Mus musculus POU domain, class 4, transcription factor 3 (Pou4f3), mRNA [NM_138945]	18998	A_55_P1997220	-2.93
Tomt	Mus musculus transmembrane O-methyltransferase (Tomt), transcript variant 2, mRNA [NM_001282088]	791260	A_55_P2121106	-2.92

Tcap	Mus musculus titin-cap (Tcap), mRNA [NM_011540]	21393	A_52_P249733	-2.86
Tmc1	Mus musculus transmembrane channel-like gene family 1 (Tmc1), mRNA [NM_028953]	13409	A_55_P2147026	-2.83
Tctex1d1	Mus musculus Tctex1 domain containing 1 (Tctex1d1), transcript variant 1, mRNA [NM_026100]	67344	A_55_P2051270	-2.82
Mei1	Mus musculus meiotic double-stranded break formation protein 1 (Mei1), transcript variant 2, mRNA [NM_001310435]	74369	A_51_P406835	-2.73
Grxcr2	Mus musculus glutaredoxin, cysteine rich 2 (Grxcr2), mRNA [NM_001033426]	332309	A_66_P106204	-2.72
Elovl2	Mus musculus elongation of very long chain fatty acids (FEN1/Elo2, SUR4/Elo3, yeast)-like 2 (Elovl2), transcript variant 2, mRNA [NM_001311121]	54326	A_55_P2062737	-2.71
Sec14l4	Mus musculus SEC14-like lipid binding 4 (Sec14l4), mRNA [NM_146013]	103655	A_51_P194853	-2.67
Slc8a2	Mus musculus solute carrier family 8 (sodium/calcium exchanger), member 2 (Slc8a2), transcript variant 1, mRNA [NM_148946]	110891	A_55_P2095563	-2.62
Ackr4	Mus musculus atypical chemokine receptor 4 (Ackr4), mRNA [NM_145700]	252837	A_52_P153291	-2.53
Palm3	Mus musculus paralemmin 3 (Palm3), mRNA [NM_028877]	74337	A_55_P2127934	-2.51
Bpifa1	Mus musculus BPI fold containing family A, member 1 (Bpifa1), mRNA [NM_011126]	18843	A_51_P202331	-2.46
Zp1	Mus musculus zona pellucida glycoprotein 1 (Zp1), mRNA [NM_009580]	22786	A_55_P2123626	-2.42
9530056K15Rik	9530056K15Rik RIKEN cDNA 9530056K15 gene	320650	A_55_P2360271	-2.41
Acbd7	Mus musculus acyl-Coenzyme A binding domain containing 7 (Acbd7), mRNA [NM_030063]	78245	A_55_P2053324	-2.34
Klhl14	Mus musculus kelch-like 14 (Klhl14), mRNA [NM_001081403]	225266	A_55_P2048085	-2.26
Ackr4	AY072938 chemokine receptor CCX CKR (exp=-1; wgp=0; cg=0), complete [TC1596586]	252837	A_55_P2050226	-2.21
Col8a2	Mus musculus collagen, type VIII, alpha 2 (Col8a2), mRNA [NM_199473]	329941	A_55_P1960631	-2.21
Lmod3	Mus musculus leiomodin 3 (fetal) (Lmod3), mRNA [NM_001081157]	320502	A_51_P427964	-2.21
Gm1043	predicted gene 1043 [Source:MGI Symbol; Acc:MGI:2685889] [ENSMUST00000213782]	381634	A_66_P139530	-2.16
Clrn2	Mus musculus clarin 2 (Clrn2), mRNA [NM_001163317]	624224	A_55_P2057806	-2.10
Pcp4	Mus musculus Purkinje cell protein 4 (Pcp4), mRNA [NM_008791]	18546	A_51_P253984	-2.09

Supplementary Table S4. Gene ontological categories of downregulated genes with statistical significance (P < 0.05) in the cochlea RNA of DT+ *Prestin-hDTR* mice.

GO accession no.	GO term	*P*-value	Gene Symbol
GO:0007605	Sensory perception of sound	8.11E-11	*Barhl1*, *Chrna9*, *Chrna10*, *Grxcr2*, *Myo15*, *Pjvk, Pou4f3, Strc, Tmc1, Tomt*
GO:0050954	Sensory perception of mechanical stimulus	2.26E-10	*Barhl1*, *Chrna9*, *Chrna10*, *Grxcr2*, *Myo15*, *Pjvk, Pou4f3, Strc, Tmc1, Tomt*
GO:0048839	Inner ear development	1.74E-08	*Chrna9*, *Chrna10*, *Gfi1*, *Myo15*, *Pou4f3, Strc, Tcap, Tmc1, Tomt*
GO:0043583	Ear development	4.74E-08	*Chrna9*, *Chrna10*, *Gfi1*, *Myo15*, *Pou4f3, Strc, Tcap, Tmc1, Tomt*
GO:0042472	Inner ear morphogenesis	3.06E-07	*Chrna9*, *Chrna10*, *Gfi1*, *Myo15*, *Pou4f3, Strc, Tcap*
GO:0042471	Ear morphogenesis	1.01E-06	*Chrna9*, *Chrna10*, *Gfi1*, *Myo15*, *Pou4f3, Strc, Tcap*
GO:0007423	Sensory organ development	3.48E-06	*Gfi1*, *Pou4f3*, *Strc*, *Tmc1*, *Tomt*
GO:0042491	Inner ear auditory receptor cell differentiation	3.48E-06	*Chrna9*, *Chrna10*, *Col8a2*, *Gfi1*, *Myo15*, *Pou4f3, Strc*, *Tcap*, *Tmc1*, *Tomt*
GO:0090596	Sensory organ morphogenesis	4.45E-06	*Chrna9*, *Chrna10*, *Col8a2*, *Gfi1*, *Myo15*, *Pou4f3, Strc*, *Tcap*, *Tmc1, Tomt*
GO:0035315	Hair cell differentiation	4.91E-06	*Gfi1*, *Pou4f3*, *Strc*, *Tmc1*, *Tomt*
GO:0042491	Inner ear auditory receptor cell differentiation	3.48E-06	*Chrna9*, *Chrna10*, *Strc*, *Tmc1*
GO:0050910|0009592|0055128	Detection of mechanical stimulus involved in sensory perception of sound	4.91E-06	*Chrna9*, *Chrna10*, *Strc*, *Tmc1*
GO:0060563	Neuroepithelial cell differentiation	1.50E-05	*Gfi1*, *Pou4f3*, *Strc*, *Tmc1*, *Tomt*
GO:0050982	Detection of mechanical stimulus	1.52E-05	*Chrna9*, *Chrna10*, *Strc*, *Tcap*, *Tmc1*
GO:0009612	Response to mechanical stimulus	2.81E-05	*Chrna9*, *Chrna10*, *Gfi1*, *Strc*, *Tcap*, *Tmc1*
GO:0060113	Inner ear receptor cell differentiation	3.05E-05	*Gfi1*, *Pou4f3*, *Strc*, *Tmc1*, *Tomt*
GO:0042490	Mechanoreceptor differentiation	4.44E-05	*Gfi1*, *Pou4f3*, *Strc*, *Tmc1*, *Tomt*
GO:0048562	Embryonic organ morphogenesis	1.52E-04	*Chrna9*, *Chrna10*, *Gfi1*, *Myo15*, *Pou4f3, Strc, Tcap*
GO:0050974	Detection of mechanical stimulus involved in sensory perception of sound	1.58E-04	*Chrna9*, *Chrna10*, *Strc*, *Tmc1*
GO:0002065	Columnar/cuboidal epithelial cell differentiation	1.95E-04	*Gfi1*, *Pou4f3*, *Strc*, *Tmc1*, *Tomt*
GO:0009581	Detection of external stimulus	3.86E-04	*Chrna9*, *Chrna10*, *Strc*, *Tcap*, *Tmc1*
GO:0009582	Detection of abiotic stimulus	3.86E-04	*Chrna9*, *Chrna10*, *Strc*, *Tcap*, *Tmc1*

GO:0048568	Embryonic organ development	0.0015099	*Chrna9*, *Chrna10*, *Gfi1*, *Myo15*, *Pou4f3, Strc, Tcap*
GO:0009913|0043355	Epidermal cell differentiation	0.0015099	*Gfi1*, *Pou4f3*, *Strc*, *Tmc1*, *Tomt*
GO:0060117	Auditory receptor cell development	0.0020491	*Strc*, *Tmc1*, *Tomt*
GO:0098858	Actin-based cell projection	0.0027265	*Bpifa1*, *Grxcr2*, *Myo15*, *Strc*, *Tmc1*
GO:0009605	Response to external stimulus	0.0027265	*Ackr4*, *Bpifa1*, *Chrna9*, *Chrna10*, *Gfi1*, *Lhx3*, *Palm3, Pou4f3, Strc, Tcap, Tmc1*
GO:0048598|0048828	Embryonic morphogenesis	0.0086246	*Chrna9*, *Chrna10*, *Gfi1*, *Myo15*, *Pou4f3*, *Strc, Tcap*
GO:0030240	Skeletal muscle thin filament assembly	0.0103704	*Lmod3*, *Tcap*
GO:0032420	Stereocilium	0.0107737	*Myo15*, *Strc*, *Tmc1*
GO:0008544	epidermis development	0.012116	*Gfi1*, *Pou4f3*, *Strc*, *Tmc1*, *Tomt*
GO:0003008	System process	0.012116	*Barhl1*, *Chrna9*, *Chrna10*, *Dfnb59*, *Grxcr2*, *Lmod3*, *Myo15*, *Pou4f3*, *Slc8a2*, *Strc*, *Tcap*, *Tmc1*, *Tomt*
GO:0009887	Animal organ morphogenesis	0.0151258	*Chrna9*, *Chrna10*, *Col8a2*, *Gfi1*, *Myo15*, *Pou4f3*, *Strc*, *Tcap*
GO:0002066	Columnar/cuboidal epithelial cell development	0.0155943	*Strc*, *Tmc1*, *Tomt*
GO:0060119	Inner ear receptor cell development	0.0155943	*Strc*, *Tmc1*, *Tomt*
GO:0032421	Stereocilium bundle	0.0155943	*Myo15*, *Strc*, *Tmc1*
GO:0014866	Skeletal myofibril assembly	0.0155943	*Lmod3*, *Tcap*
GO:0048513	Animal organ development	0.0325121	*Barhl1*, *Chrna9*, *Chrna10*, *Col8a2*, *Gfi1*, *Lhx3*, *Lmod3*, *Myo15*, *Pou4f3*, *Strc*, *Tcap*, *Tmc1*, *Tomt*
GO:0032426	Stereocilium tip	0.0334263	*Strc*, *Tmc1*
GO:0050877	Nervous system process	0.0373343	*Barhl1*, *Chrna9*, *Chrna10*, *Dfnb59*, *Grxcr2*, *Myo15*, *Pou4f3*, *Slc8a2*, *Strc*, *Tmc1*, *Tomt*
GO:0007600	Sensory perception	0.0376413	*Barhl1*, *Chrna9*, *Chrna10*, *Dfnb59*, *Grxcr2*, *Myo15*, *Pou4f3*, *Strc*, *Tmc1*, *Tomt*
GO:0048699	Generation of neurons	0.0475618	*Barhl1*, *Gfi1*, *Lhx3*, *Ocm*, *Pcp4*, *Pou4f3*, *Strc*, *Tmc1*, *Tomt*

Supplementary Table S5. Primers used for qRT-PCR analysis in this study.

Gene	Forward primer (5'→3')	Reverse primer (5'→3')	Reference
Apoc2	GCATGGATGAGAAACTCAGGG	AAAATGCCTGCGTAAGTGCTC	4
Barhl1	CAAAGTGAAGGAGGAGGGCG	GTGTCGGTGAGGTTGAGCGA	5
Ccl2	GAGGAAGGCCAGCCCAGCAC	TGGGGCGTTAACTGCATCTGGC	6
Ccl4	GCCCTCTCTCTCCTCTTGCT	CTGGTCTCATAGTAATCCATC	7
Ccl7	TGTCCCTGGGAAGCTGTTAT	CTTTGGAGTTGGGGTTTTCA	This study
Cd28	GCCTTACCTAGACAACGAGA	CCAAAACAGCTTAGGAGATGAC	This study
Cdsn	CTGATGGCCGGTCTTATTCT	GCTGTTGGAGCCAGTCTTTC	8
Chrna9	GTCCCTCTGATAGGAAAATACTAC	CTAAGGCAGCTCTCACCCAC	This study
Chrna10	GTTTCGTGACCTGTTTGCCAACT	GGTACAAGGTCAGCACTTGGTTC	This study
Cldn22	GGGCTTAGTCTTCCGAACG	CTTCCAGTGCGGCAAGTAGT	This study
Gapdh	TCGGTGTGAACGGATTTG	CGTGAGTGGAGTCATACTGGA	This study
Gfi1	GAGCAACACAAGGCAGTG	TCTTGCCACAGATCTTACAGTC	9
Grxcr2	CCAGCCTCTGTTCAATGACTAC	CCTCATGAAGTCCCTTTTGTCC	This study
Lgals3	CAACGCAAACAGGATTGTTC	CGTGTTACACACAATGACTCTCC	10
Lhx3	CCCCCACCCATGAGGGTGCT	GAGCCAGGGGAAGCAGAGGC	11
Loxl4	CCGCTGCAAGTATGATGG	GTTCCTGAGTCGCTGTTC	12
Mpeg1	GTGAAACAAAAGCCAGACAGAGCCT	TCATGGCGCAGATGGTTTTGGC	13
Myo15	ACCAACAGATGCTCACTGTCTCTG	ATAGAAGGCAGGTTGCGTATGGAG	This study
Ocm	AGACAACG CCAGAGTGGATAC	TCAAGGACTTGGTCTCTGACTC	This study
Pjvk	TTCATCTACCTGGACGGTGCCTT	TCCAGGTAAAGCTGGGAACGAGA	This study
Pou4f3	ATGCGCCGAGTTTGTCTCC	GGGCTTGAACGGATGATTCT	14
Sirpb1b	GTCACTCCTGCTGATTCGG	GTCACTGTCTGCTGAGGGAC	This study
Slc26a5	TGTTCCTCGGCTTGGACTAC	GTCAATGTACACATCCGTGTCAG	This study
Sox2	ACTTTTGTCCGAGACCGAGAA	CGCGGCCGGTATTTATAATC	15
Strc	CCTGACGCTCATGCTTCGTTC	CAGCCAGGACACTGTCGTTG	This study
Tmc1	CATCTGCAGCCAACTTTGGTGTGT	AGAGGTAGCCGGAAATTCAGCCAT	16
Tomt	ACTTCGGATTGAGGAGCGGGCTTT	ACCAGCCGCATCAGAATCTGACCT	This study
Vgf	TCGACCATCGCTCATACTCC	CGTGAAGGTTTTCATGACCAAC	This study
Vglut3	TGGTGCTTGCTGTAGGATTTA	CCACGCCATTTGAGATCC	17
Wnt2	CACCCTGGACAGAGATCACA	ACAACGCCAGCTGAAGAGAT	18

Supplementary references

1 Drouin, R., Therrien, J. P., Angers, M. & Ouellet, S. In vivo DNA analysis. *Methods in Molecular Biology (Clifton, N.J.)* **148**, 175-219, doi:10.1385/1-59259-208-2:175 (2001).

2 Ensembl Genome Browser, last Update. October, 2018.

3 Acar-Perk, B. *et al.* The t(14,15) in mouse strain CBA/CaH-T(14;15)6Ca/J causes a break in the ADAMTS12 gene. *Comparative medicine* **60**, 118-122 (2010).

4 Cai, Y. *et al.* Mice lacking prostaglandin E receptor subtype 4 manifest disrupted lipid metabolism attributable to impaired triglyceride clearance. *FASEB J* **29**, 4924-4936, doi:10.1096/fj.15-274597 (2015).

5 Li, S. Barhl1 Regulates Migration and Survival of Cerebellar Granule Cells by Controlling Expression of the Neurotrophin-3 Gene. *Journal of Neuroscience* **24**, 3104-3114, doi:10.1523/jneurosci.4444-03.2004 (2004).

6 Hildebrand, D. G. *et al.* IkappaBzeta is a transcriptional key regulator of CCL2/MCP-1. *J Immunol* **190**, 4812-4820, doi:10.4049/jimmunol.1300089 (2013).

7 Kim, W. *et al.* Hippo signaling interactions with Wnt/beta-catenin and Notch signaling repress liver tumorigenesis. *J Clin Invest* **127**, 137-152, doi:10.1172/JCI88486 (2017).

8 Leclerc, E. A. *et al.* Corneodesmosin gene ablation induces lethal skin-barrier disruption and hair-follicle degeneration related to desmosome dysfunction. *J Cell Sci* **122**, 2699-2709, doi:10.1242/jcs.050302 (2009).

9 Bjerknes, M. & Cheng, H. Cell Lineage metastability in Gfi1-deficient mouse intestinal epithelium. *Dev Biol* **345**, 49-63, doi:10.1016/j.ydbio.2010.06.021 (2010).

10 Hollern, D. P., Honeysett, J., Cardiff, R. D. & Andrechek, E. R. The E2F transcription factors regulate tumor development and metastasis in a mouse model of metastatic breast cancer. *Mol Cell Biol* **34**, 3229-3243, doi:10.1128/MCB.00737-14 (2014).

11 Son, E. Y. *et al.* Conversion of mouse and human fibroblasts into functional spinal motor neurons. *Cell Stem Cell* **9**, 205-218, doi:10.1016/j.stem.2011.07.014 (2011).

12 Li, R. K. *et al.* Lysyl oxidase-like 4 (LOXL4) promotes proliferation and metastasis of gastric cancer via FAK/Src pathway. *J Cancer Res Clin Oncol* **141**, 269-281, doi:10.1007/s00432-014-1823-z (2015).

13 Erener, S. *et al.* ARTD1 deletion causes increased hepatic lipid accumulation in mice fed a high-fat diet and impairs adipocyte function and differentiation. *FASEB J* **26**, 2631-2638, doi:10.1096/fj.11-200212 (2012).

14 Chonko, K. T. *et al.* Atoh1 directs hair cell differentiation and survival in the late embryonic mouse inner ear. *Dev Biol* **381**, 401-410, doi:10.1016/j.ydbio.2013.06.022 (2013).

15 Narayana, Y. V., Gadgil, C., Mote, R. D., Rajan, R. & Subramanyam, D. Clathrin-Mediated Endocytosis Regulates a Balance between Opposing Signals to Maintain the Pluripotent State of Embryonic Stem Cells. *Stem Cell Reports*, doi:10.1016/j.stemcr.2018.11.018 (2018).

16 Kawashima, Y. *et al.* Mechanotransduction in mouse inner ear hair cells requires

transmembrane channel-like genes. *J Clin Invest* **121**, 4796-4809, doi:10.1172/JCI60405 (2011).

17 Lau J. C., Kroes R. A., Moskal J. R., Linsenmeier R. A. Diabetes changes expression of genes related to glutamate neurotransmission and transport in the Long-Evans rat retina. *Mol Vis* **19**, 1538-1553 (2013).

18 Zhang, A., Shen, C. H., Ma, S. Y., Ke, Y. & El Idrissi, A. Altered expression of Autism-associated genes in the brain of Fragile X mouse model. *Biochem Biophys Res Commun* **379**, 920-923, doi:10.1016/j.bbrc.2008.12.172 (2009).
